# Gender Justice in the Triple Planetary Crisis: A Scoping Review

**DOI:** 10.1111/jan.70620

**Published:** 2026-05-09

**Authors:** Catelyn Richards, Micah Peters, Julia Charalambous, Lila Singh‐Peterson, Lucy Osborn, Aletha Ward, Jessica LeClair, Tracy Levett‐Jones

**Affiliations:** ^1^ University of Technology Sydney Sydney Australia; ^2^ Australian Nursing and Midwifery Federation (ANMF), Federal Office Melbourne Australia; ^3^ Rosemary Bryant AO Research Centre, College of Health Adelaide University Adelaide South Australia Australia; ^4^ Health Evidence Synthesis, Recommendations and Impact (HESRI), School of Public Health, College of Health Adelaide University Adelaide South Australia Australia; ^5^ The Danish Centre of Systematic Reviews: A Joanna Briggs Institute Centre of Excellence, Department of Clinical Medicine Aalborg University Aalborg Denmark; ^6^ Office of the Pro‐Vice Chancellor (First Nations Strategy) University of Southern Queensland Ipswich Queensland Australia; ^7^ School of Nursing and Midwifery Edith Cowan University Joondalup Western Australia Australia; ^8^ Climate Action Nurses Brisbane Queensland Australia; ^9^ University of Southern Queensland Toowoomba Queensland Australia; ^10^ Federation University Melbourne Australia; ^11^ University of Wisconsin‐Madison Madison Wisconsin USA

**Keywords:** biodiversity loss, climate change, equity, gender justice, nurses, pollution, triple planetary crisis

## Abstract

**Aim:**

To identify and report how gender justice is conceptualised and discussed in contemporary health literature in relation to the Triple Planetary Crisis of climate change, pollution and biodiversity loss, with a particular focus on the experiences of women and gender‐diverse populations, and the representation of nurses and other healthcare professionals dominated by women.

**Design:**

Scoping review.

**Data Sources:**

Searches were conducted across MEDLINE (Ovid), Scopus, CINHAL, Embase and ProQuest, focusing on studies published from January 1 2000–23 September 2024.

**Review Methods:**

The review was conducted in accordance with the JBI methodology for scoping reviews and reported against the Preferred Reporting Items for Systematic Reviews and Meta‐Analyses extension for Scoping Reviews (PRISMA‐ScR) guidelines. Data were extracted according to a pre‐specified extraction framework, developed a priori, encompassing components of gender justice and intersectionality.

**Results:**

A total of 39 studies were included: 17 (43.6%) qualitative, 17 (43.6%) quantitative and 5 (12.8%) mixed methods. The focus of the studies included gendered experiences of climate change (30.8%), decision‐making and governance (20.5%), health and wellbeing (17.9%), women's economic participation (15.4%), cultural and spiritual connections to land (7.7%), and intersectionality and human rights (7.7%). Gender‐diverse populations, nurses and other healthcare professions dominated by women were not represented in the literature.

**Conclusion:**

The literature reported that women experienced differentiated exposure to the Triple Planetary Crisis. The underrepresentation of gender diverse people and nurses in recent studies remains a significant barrier to advancing understanding of gender justice. Integrating gender justice into health systems is increasingly important to prevent women from being disproportionately impacted by the Triple Planetary Crisis.

**Impact:**

More attention to inclusion of frontline healthcare professionals, including nurses, in governance, policy discussions and leadership roles could strengthen the response to systemic environmental threats.

**Patient or Public Contribution:**

No patient or public involvement.

## 1 Introduction

The interconnected crises of climate change, biodiversity loss and pollution are collectively termed the Triple Planetary Crisis (Passarelli et al. 2021; United Nations Environment Programme 2021). These are accelerating global ecological collapse, with profound consequences for human health. The impacts of the Triple Planetary Crisis are not evenly distributed. Persistent inequities mean women and gender‐diverse populations are disproportionately affected and often excluded from decision‐making processes that shape their futures (Solar and Irwin 2010; World Health Organization 2024). Similar inequities are evident within the health sector, where gender continues to shape the professional landscape broadly, and nursing in particular, as a highly gendered profession (Langer et al. 2015).

This scoping review examines how gender justice, broadly defined as the pursuit of equity and equality through a gendered lens, (UN Women 2010) is conceptualised in the international healthcare literature concerning the Triple Planetary Crisis. The review focuses on the experiences of women and gender‐diverse populations, and the representation of nurses and other healthcare professionals dominated by women in the extant literature on this topic.

### 1.1 Background

#### 1.1.1 Beyond the Tipping Point A Planet in Crisis

Across the planet, the stability of interconnected ecological systems and human and non‐human systems—collectively referred to as ‘more‐than‐human’—are being disrupted by unprecedented levels of environmental degradation. Among the most pressing of these threats are climate change, biodiversity loss and pollution (the Triple Planetary Crisis), which collectively undermine the foundations for all life on Earth (Passarelli et al. 2021; United Nations Environment Programme 2021).

#### 1.1.2 The Triple Planetary Crisis

The scientific evidence reveals a rapidly changing Earth system that is moving beyond safe environmental limits that support human life (Caesar et al. 2024; Rockström et al. 2009; Steffen et al. 2015). Greenhouse gas emissions continue to rise beyond safe limits trapping heat (Intergovernmental Panel on Climate Change 2023), and biodiversity is declining rapidly, as species vanish and ecosystems lose their ability to function and flourish (Romanelli et al. 2015). These problems are further exacerbated by pollution from plastics (Dimassi et al. 2022; Yates et al. 2021) and synthetic chemicals (Lin et al. 2023) along with wildfires and dust storms that reduce air quality and place further strain on living systems. Each separate, systemic environmental threat interacts and amplifies the others (Caesar et al. 2024). Land clearing, for instance, drives biodiversity loss while simultaneously decreasing the atmospheric capacity to sequester carbon dioxide emissions and filter pollutants (Roy et al. 2022). These interconnected, accelerating environmental threats not only undermine planetary stability but are causing and risk further impacts on human health and wellbeing (Whitmee et al. 2015).

The consequences of the Triple Planetary Crisis have significant and protracted health and wellbeing impacts (Romanello et al. 2024). For example, worsening air pollution perpetuated by global emissions and exacerbated by rising temperatures impacts respiratory and cardiovascular health (Porter and Heald 2019). Meanwhile, extreme heat days continue to grow in severity and frequency, placing populations, and especially pregnant people, children, outdoor workers, older people and those with comorbidities at risk of heat‐related illness and death (Romanello et al. 2024).

Biodiversity loss and extreme weather events are disrupting water supplies and increasing the risk of waterborne diseases; changing vector habitats are impacting the range of diseases like malaria and dengue fever; and food security is being compromised by altered crop yields, resulting in malnutrition and foodborne diseases (Robinson et al. 2024). The health impacts of the Triple Planetary Crisis extend beyond individual physiology, however, and collide with entrenched inequities, meaning that health burdens are not experienced homogenously across populations.

#### 1.1.3 Intersecting Inequities

The Triple Planetary Crisis also perpetuates and exacerbates existing social inequalities, disproportionately affecting particular groups. These disproportionate inequalities are not incidental but are shaped by structural, social and cultural determinants of health (Redvers et al. 2022; Solar and Irwin 2010). Social determinants, including gender norms, income, education, housing, political participation and environmental conditions, are the underlying drivers of health inequities between different population groups (Solar and Irwin 2010; World Health Organization 2024). These determinants intersect to produce cumulative disadvantage (Crenshaw 1991).

Distinct from social determinants of health, which refer to structural and socioeconomic conditions, are cultural determinants, which shape wellbeing through connection to culture, land, language, kinship systems and are equally important drivers of health (Redvers et al. 2022).

#### 1.1.4 Gender Justice

A growing body of research demonstrates that women and gender‐diverse (people whose gender identity is not exclusively male or female or whose gender identity differs from the sex they were assigned at birth) populations (van Daalen et al. 2024), particularly those from low‐resource countries and communities, face disproportionate impacts from climate change, pollution and biodiversity loss (Intergovernmental Panel on Climate Change 2023). Persistent biased social norms are recognised as a major obstacle in achieving gender equality and the empowerment of women and girls (Sustainable Development Goal 5) as they ‘constrain women's choices and opportunities by regulating behaviour and setting the boundaries of what women are expected to do and be.’ (United Nations Development Programme 2023).

Gender justice is closely linked to both intersectionality and ecofeminism, providing complementary lenses for understanding and addressing systemic inequalities. An intersectional approach as defined by Crenshaw (1991) recognizes how overlapping systems of oppression such as gender, race, class, sexuality and disability compound disadvantage. Similarly, ecofeminist perspectives connect gender justice to environmental and planetary health, highlighting how patriarchal and extractivist systems underpin both gender inequities and ecological degradation (Djoudi et al. 2016; Sultana 2022).

There are multiple definitions and interpretations of gender justice across policy, law, and feminist scholarship. UN Women (2010) defines gender justice as ‘ending the inequalities between women and men that are produced and reproduced in the family, the community, the market and the state’. Gender justice extends to recognising that dominant gender‐based social norms and biases reproduce patterns of differentiation in respect to roles and responsibilities, status and authority, voice, agency, and access to resources and opportunities (Hawkes et al. 2025). In this scoping review, we thus define gender justice as ‘the fair and equitable treatment of all genders, achieved by dismantling structural inequalities and ensuring accountability for gender‐based harms’.

#### 1.1.5 The Triple Planetary Crisis and Gender Justice

Emerging scholarship demonstrates that gendered norms shape differential exposure to climate change, pollution, and biodiversity loss, by structuring power relations that often inequitably determine who performs unpaid care labour, who controls and accesses resources, and whose knowledge and priorities are reflected in decision‐making (Global Health 50–50 2024). The World Health Organization (2024) highlights that reducing exposure to health inequities and enhancing resilience in the face of the planetary crisis requires targeted action. Addressing gender justice is therefore a critical dimension of addressing the collective environmental challenges that society presently faces. While the Triple Planetary Crisis produces deeply gendered impacts that exacerbate inequities, advancing gender justice may represent a structural pathway to address these imbalances by addressing inequity.

#### 1.1.6 A Critical Gap

Despite growing recognition of the impacts of environmental change on women and gender‐diverse populations, significant gaps remain in the evidence base connecting the domains of the Triple Planetary Crisis with gender justice. While much has been written on the topic of environmental justice and gender, relatively little research appears to have been conducted and reported. Many articles are non‐empirical commentaries, editorials or discussion papers (Afifi et al. 2024; Barkha 2022; Djoudi et al. 2016; Enarson 2013).

Based on a preliminary search of the literature, existing scoping and systematic reviews focus primarily on climate change‐related health impacts, overlooking the systemic, interconnected effects of the Triple Planetary Crisis and broader social environmental determinants of health. For example, a recent literature review conducted by Keenan et al. (2024) examined the health impacts of climate change on women; however, its focus was limited to climate change and pregnancy‐related outcomes, leaving broader systemic gender justice issues underexplored. Similarly, a scoping review by Stone et al. (2022) found that there were various mental health effects experienced by women—again within the narrowed focus of climate change. While the systematic review by Khine and Langkulsen (2023) expanded the examination of broader determinants of health and marginalised populations (including women), again, this paper was limited to climate change‐related effects.

Finally, to the authors' knowledge, it is unclear what focus, if any, has been placed on the gendered experiences of healthcare professionals, including nurses, associated with the cascading effects of the Triple Planetary Crisis. Not only are nursing and many health professions dominated by women (often around 90%) (World Health Organization 2025), nurses' frontline roles provide insight into how environmental changes differentially impact populations. Their commitment to person‐centred care equips them to advocate for inclusive and equitable responses. By integrating principles of gender justice into practice and advocacy, nurses might influence research, policy and health system transformation. This positions the profession as a vital force in advancing gender‐equitable solutions to the Triple Planetary Crisis (Hopkins‐Walsh et al. 2023).

## 2 The Review

### 2.1 Aims

This scoping review aimed to identify and report how gender justice is conceptualised and discussed in contemporary health literature in relation to the Triple Planetary Crisis of climate change, pollution and biodiversity loss, with a particular focus on the experiences of women and gender‐diverse populations and the representation of nurses and other healthcare professionals dominated by women.

It sought to answer the primary question: ‘What is known about gender justice in the context of the Triple Planetary Crisis in relation to the representation of women, gender‐diverse populations, nurses and those working in health professions, often dominated by women?’

Supplementary research questions include:

1. Which structural and systemic factors impacting gender equity have been identified?
2. Which pathways to empowerment, agency, and redistribution of power for women and gender‐diverse populations have been identified?
3. What outcomes have been reported from efforts to advance gender equity in the context of the Triple Planetary Crisis?
4. How are nurses and other health professionals, women, and gender‐diverse individuals represented in this body of literature?

## 3 Methods

### 3.1 Design

This review was conducted following the JBI methodology for scoping reviews (Peters et al. 2020). An apriori protocol for this review was prepared and registered with the Open Science Framework. This Scoping Review is reported in accordance with the PRISMA‐ScR (Preferred Reporting Items for Systematic Reviews and Meta‐Analyses Extension for Scoping Reviews) checklist (Tricco et al. 2018).

### 3.2 Search Methods

Following an initial search to identify any preexisting relevant reviews and to establish search terms and concepts, a comprehensive search was conducted across MEDLINE (Ovid), Scopus, CINAHL, Embase, and ProQuest on 23 September 2024, focusing on studies published 2000–23 September 2024. Search terms were initially discussed by the entire research team and developed by identifying key terms related to the overarching columns ‘Women and Gender diverse people’, and ‘The Triple Planetary Crisis’. Free text search and subject headings were included under each column. Search terms were then piloted in MEDLINE (Ovid) and refined with assistance from a senior research librarian (Scoping Review Question and Analytical FrameworkKP). Given how extensive the subject topic is, over 48 search terms were used to search databases. The MEDLINE (Ovid) database search strategy has been presented in Appendix A. This was adapted for the other databases as necessary.

### 3.3 Inclusion and Exclusion Criteria

Following the JBI recommendation for scoping reviews, the Participants–Concept–Context (PCC) framework was applied to develop inclusion/exclusion criteria and guide the selection of studies.

#### 3.3.1 Participants

Participants in the studies included women and gender‐diverse people as well as health professionals including nurses. We adopted an inclusive understanding of ‘women and gender‐diverse people’ to encompass all individuals who self‐identify as women, as well as those whose gender identity falls outside the binary. Gender itself refers to a socially and culturally constructed set of roles, behaviours and identities, distinct from biological sex and inclusive of diverse experiences such as transgender and non‐binary gender identities. Studies involving mixed populations were included in the review where data relating specifically to women and gender‐diverse people could be disaggregated and meaningfully interpreted. Definitions of women and gender‐diverse people are provided in the protocol.

#### 3.3.2 Concept

To be eligible, sources must include information in relation to at least one element of the Triple Planetary Crisis and gender justice. Further details of the conceptual framework and definitions for these concepts are explained in the review protocol.

#### 3.3.3 Context

The review adopted a broad inclusion of sources and did not restrict the inclusion of sources based on country or field of research.

#### 3.3.4 Sources of Evidence

Studies of qualitative, quantitative, and mixed methods methodologies were included in the review. In line with the objectives of this scoping review, the search strategy was restricted to peer‐reviewed empirical literature. Grey literature was not included, as the review sought to map how planetary health concepts have been empirically investigated within the health literature, rather than to capture policy, educational resources or non‐peer‐reviewed commentary. The year 2000 represents a critical juncture in global environmental and gender equity efforts. It marks the launch of the Millennium Development Goals, which positioned gender equality as a global priority, and coincided with a growing shift in environmental discourse that began to frame ecological degradation as a human rights and justice issue. As such, to maintain contemporary relevance, only papers published after 1 January 2000 were considered, with older publications excluded.

### 3.4 Source Screening and Selection

The Source Screening and Selection was performed in Covidence Software (Covidence 2024). This screening was conducted independently by two researchers (x and x), with any conflicts resolved by a third researcher (x). Articles retained from this then underwent full text retrieval and screening. Full text screening was performed by two researchers (x and x) with disagreements resolved by a third researcher (x).

The search retrieved 2768 articles. Figure 1 reports the screening process that was undertaken following the Preferred Reporting for Systematic Reviews and Meta‐Analyses extension for Scoping reviews (PRISMA‐ScR) method (Peters et al. 2020). After 309 articles were removed (292 automated through Covidence and reference management software and 17 manually). There were 2459 articles retrieved which were title and abstract screened against the study aims and objectives, with 2276 excluded during this phase. Eight records identified as potentially relevant could not be retrieved in full text despite reasonable efforts within the constraints of the review timeline. These records were therefore excluded prior to the full‐text screening stage. 183 were sought for full text retrieval. 175 articles were then assessed for their eligibility. 136 articles were removed for various reasons, including wrong study design/methodology (*n* = 53), the study did not focus on gender justice‐related themes (*n* = 49), the context of the study was not planetary health/the Triple Planetary Crisis (*n* = 31), or the English full text was unavailable (*n* = 3). Data was then extracted from the remaining 39 articles. All exclusions were documented with a rationale into Covidence (Covidence 2024).

**FIGURE 1:**
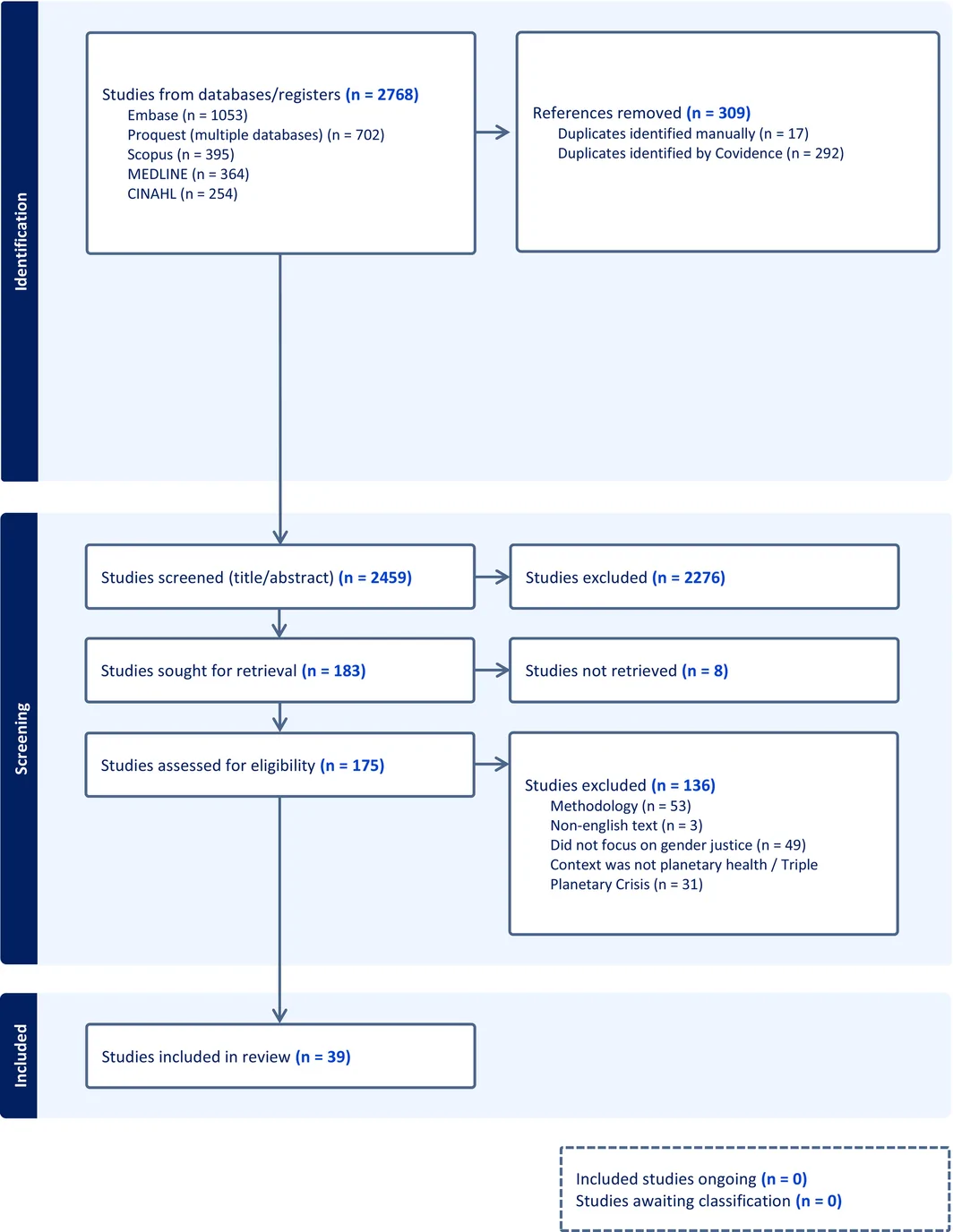
PRISMA.

### 3.5 Critical Appraisal

The Mixed Methods Appraisal Tool (MMAT) (Hong et al. 2018) was applied for standardised appraisal across dynamic studies and to enhance rigour. However, in keeping with JBI scoping review methodology and the PRISMA‐ScR reporting guideline, no papers were excluded based on the MMAT score. Likewise, the authors did not modulate interpretation of the included studies' findings based on the results of quality appraisal.

### 3.6 Data Extraction

#### 3.6.1 Data Abstraction

Prior to data extraction, two researchers (x and x) read and synthesised pertinent theory from the fields of gender justice, ecofeminism, and intersectionality to establish a data extraction template relevant to the research aims. This template was guided by theoretical concepts from the fields of intersectionality and ecofeminism to inform the categorisation of data in relation to gender justice (D1‐D6) (Table One). Once reviewed and refined by the entire research team, the framework formed the deductive codebook used to guide the extraction of relevant data and identification of key themes from the retained articles. Full texts were uploaded into NVivo Version 14 (QSR International 2023) and Covidence (Covidence 2024) software, which were used to extract the data using the pre‐established extraction template. Rationales for including findings from each text were recorded as top‐level ‘codes’, which consolidated related findings into broader categories.

In keeping with Peters et al. (2020), the extracted data were analysed descriptively. Frequency counts, sourced from NVivo, were used to summarise key concepts, populations and study locations.

#### 3.6.2 Synthesis

The findings were initially mapped against the extraction framework (Table 1) and then discussed with the research team. Consensus on the extracted data was reached through discussion, comparison, and synthesis. The results are presented in a summary table outlining study characteristics and Figures (1, 2, 3, 4, 5, 6) to illustrate the distribution and analytic focus of the included literature. Figures are based on NVivo coding frequency counts. ‘Number of studies’ refers to the number of unique articles in which a theme was identified at least once. ‘Times referenced’ refers to the total number of coding references across all included studies. Themes are not mutually exclusive, and a single study may contribute multiple references to one or more themes.

**TABLE 1:** Scoping Review Question and Analytical Framework.

Aspect/Research question	Aligned extraction theme (D1‐D8)
Study Characteristics	Author, year, title, location, study aims, study design, methods, population, intervention/phenomena of interest, findings/conclusion and limitations. *Domain 1* (D1)—Cultural Context: HDI and GDI.
Critical appraisal	Mixed Methods Assessment Tool used to critique the papers and score out of 5.
*Sup Question 1*: Which structural and systemic factors impacting gender equity have been identified?	*Domain 2* (*D2*)—Systemic oppression and power dynamics: colonialism, capitalism, patriarchy, and other intersecting systems that perpetuate gender inequity. *Domain 3* (*D3*)—Marginalisation and disproportionate impacts: differential exposure, vulnerability, and access to resources.
*Sup Question 2*: Which pathways to empowerment, agency, and redistribution of power for women and gender‐diverse populations have been identified? *Sup Question 3*: What outcomes have been reported from efforts to advance gender equity in the context of the Triple Planetary Crisis?	*Domain 4* (*D4*)—Pathways toward gender justice: Representation in decision‐making and reversal of dominant Global North narratives. *Domain 5* (*D5*)—Outcomes of gender equity: Impacts of empowerment and leadership.
*Sup Question 4*: How are nurses and other health professionals, women, and gender‐diverse individuals represented in this body of literature?	*Domain 6* (*D6*)—Health professionals: experiences, representations, and absence in gender justice discourse within the Triple Planetary Crisis.

**FIGURE 2:**
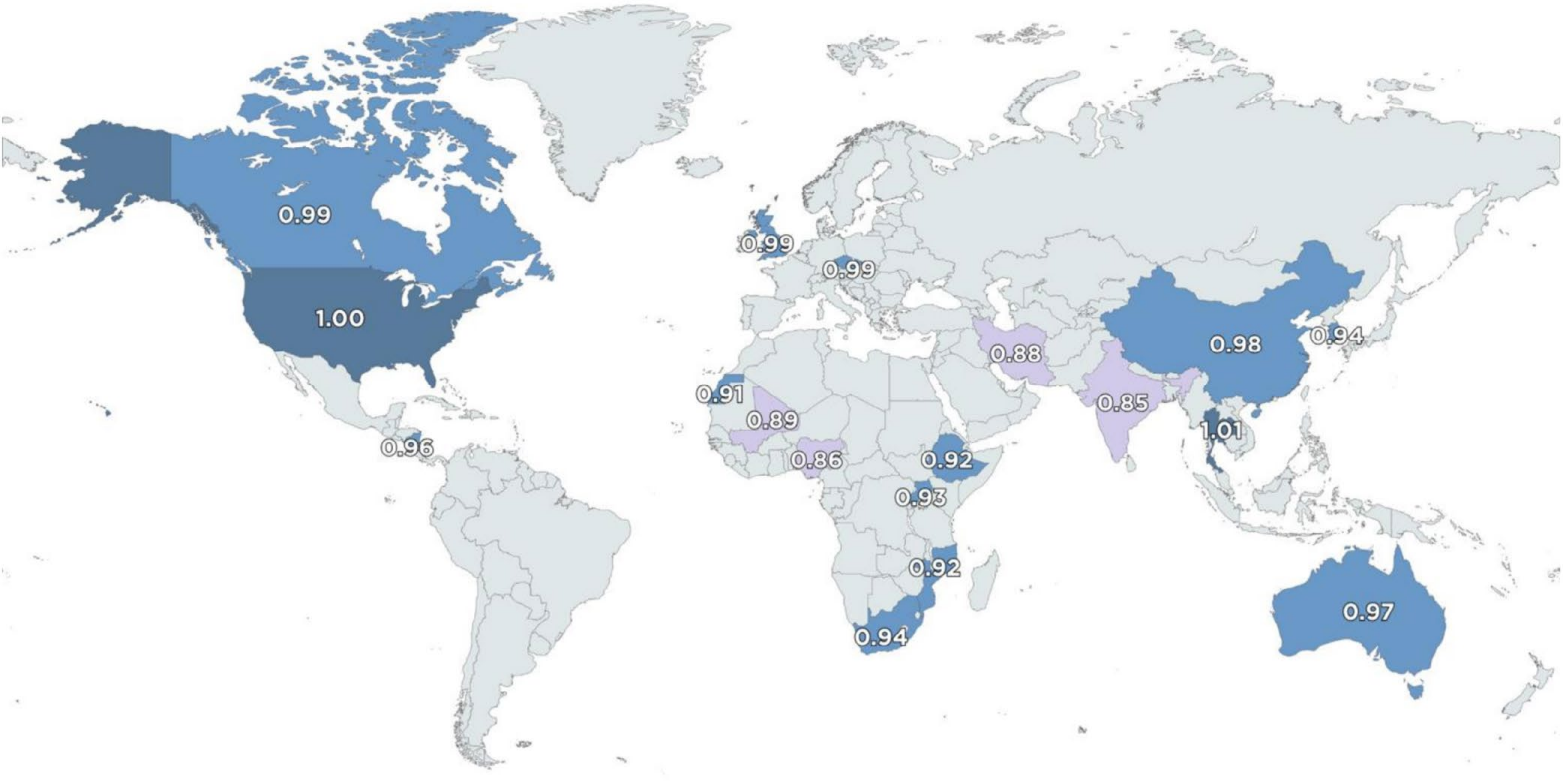
Gender development index map.

**FIGURE 3:**
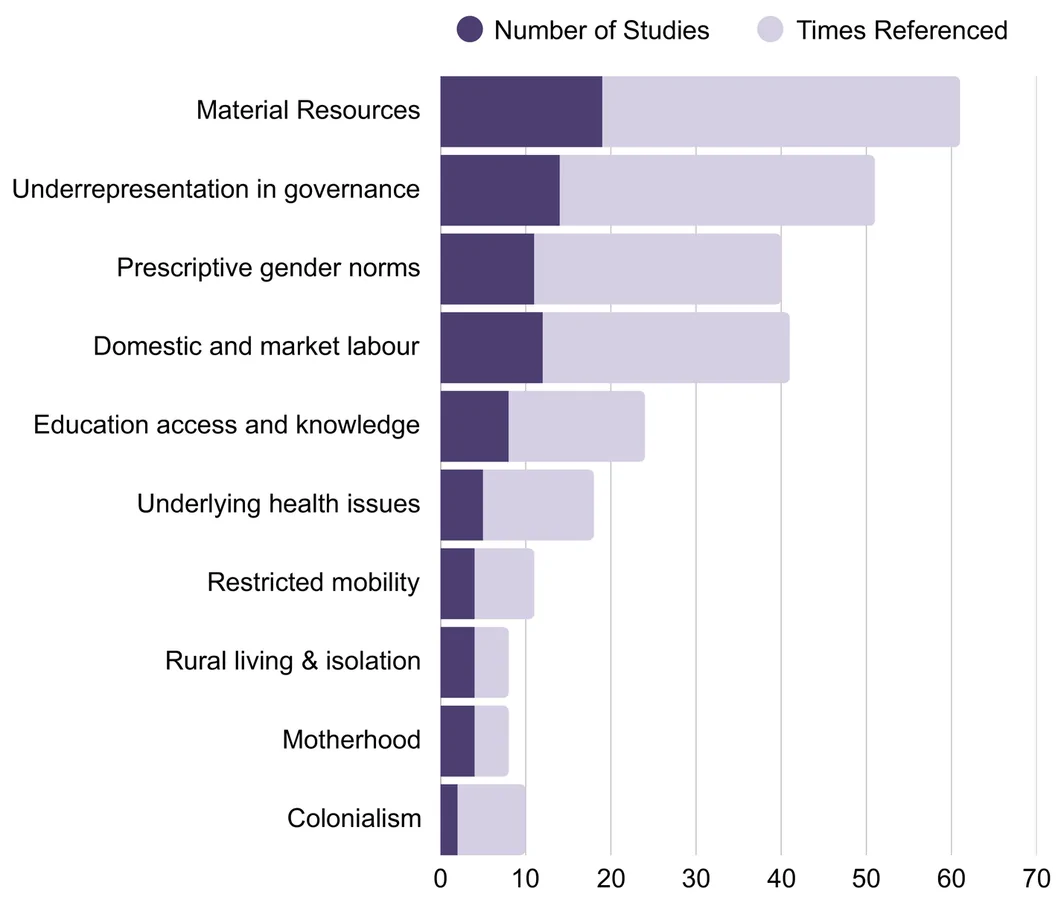
Factors that contribute to the systemic oppression of women reported in the literature.

**FIGURE 4:**
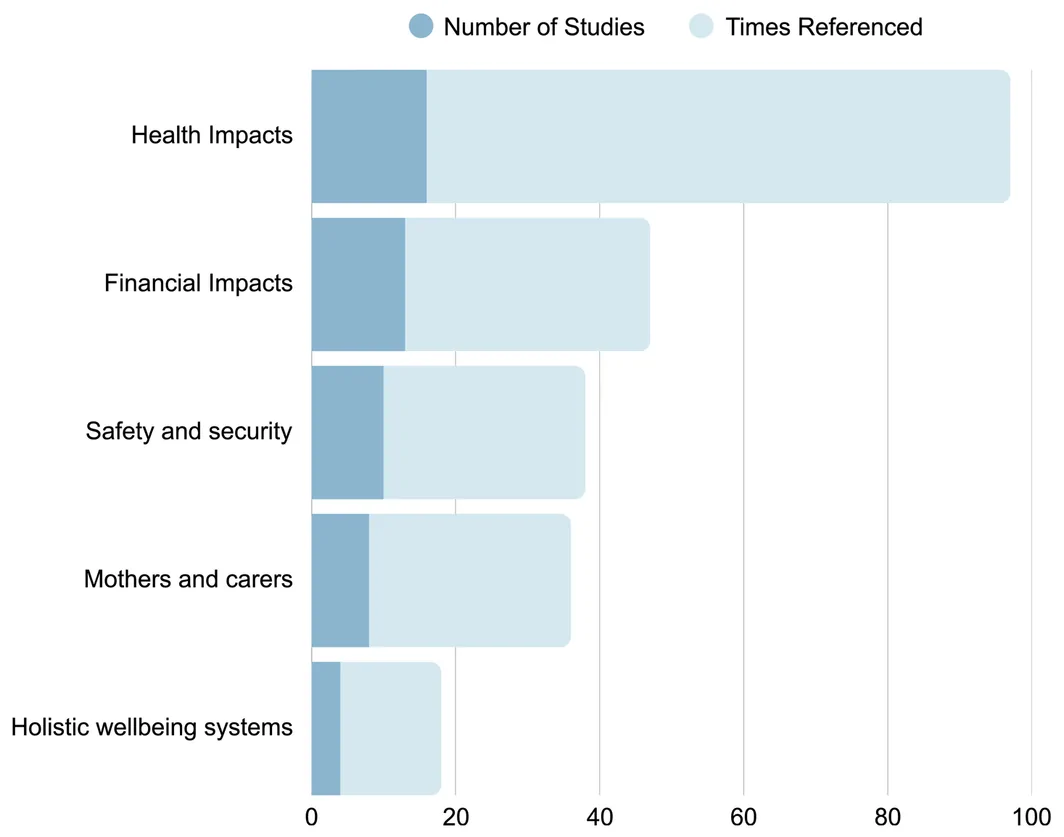
Examples of Triple Planetary Crisis impacts on women reported in the literature.

**FIGURE 5:**
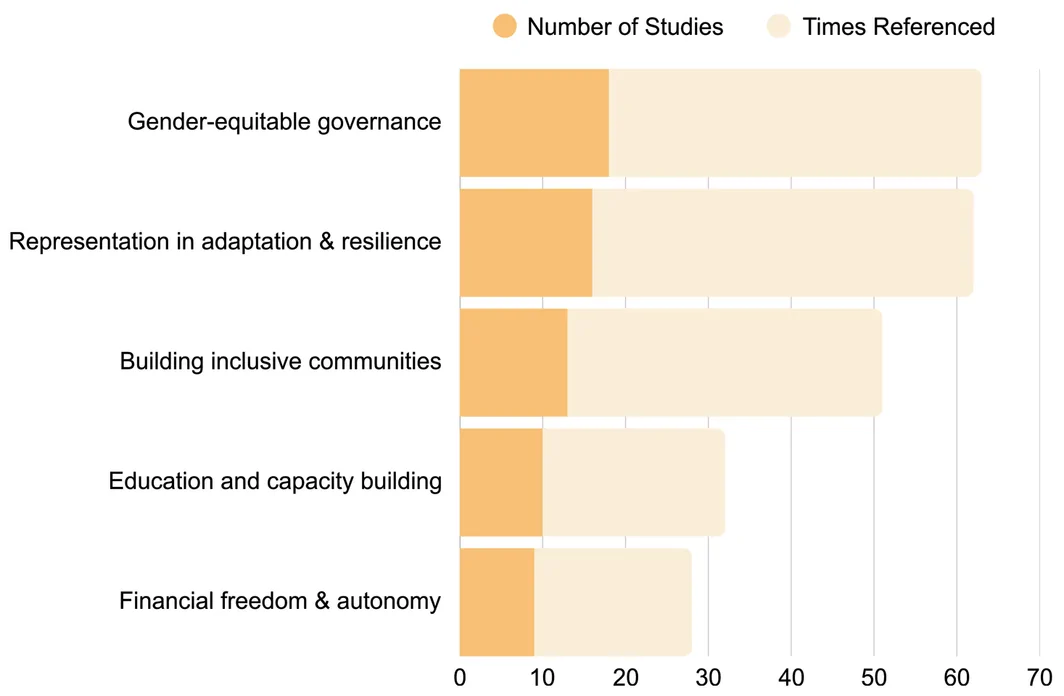
Examples of pathways toward gender justice reported in the literature.

**FIGURE 6:**
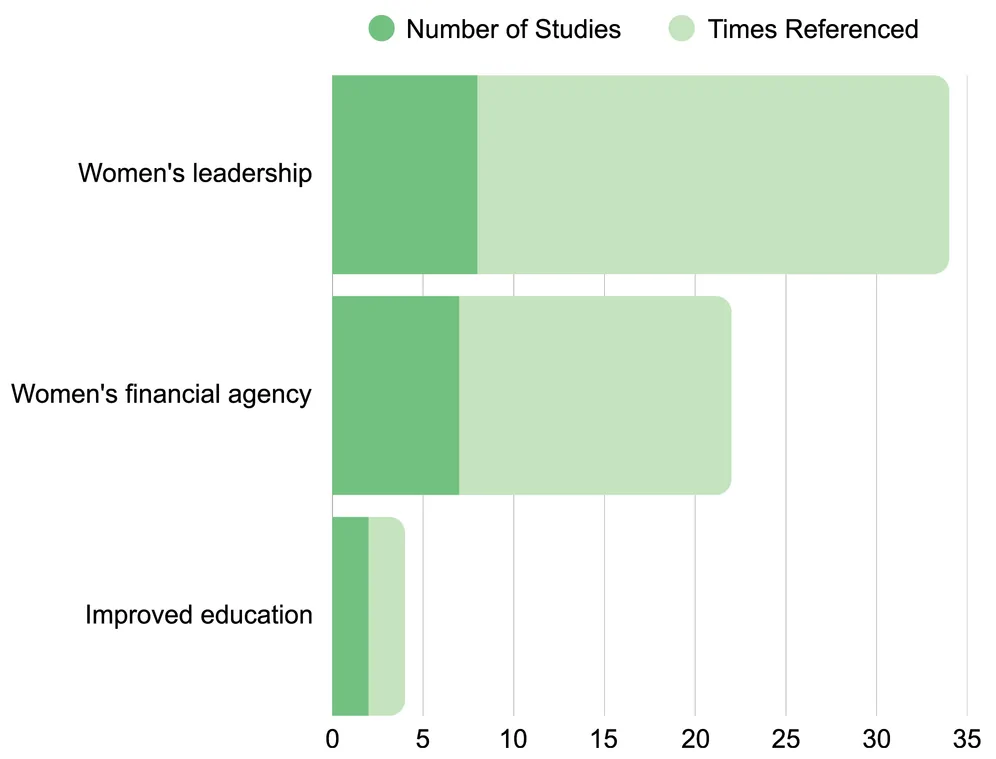
Outcomes reported in the literature.

## 4 Results

### 4.1 Study Characteristics

#### 4.1.1 Study Design

Of the 39 included studies, 17 (43.6%) employed qualitative designs, 17 (43.6%) were quantitative, and 5 (12.8%) used mixed methods approaches. Within the qualitative studies, 10 (25.6%) used interviews, 4 (10.3%) adopted case study designs, and 3 (7.7%) employed ethnographic or field research methods. Quantitative studies were primarily cross‐sectional analyses (9, 23.1%) or econometric/modelling studies (7, 17.9%), with one (2.6%) using an ecological time‐series design. Mixed‐methods studies (5, 12.8%) combined qualitative techniques such as interviews or focus groups with quantitative surveys or statistical analyses to provide a more comprehensive understanding of the phenomena investigated.

#### 4.1.2 Phenomena of Interest

The phenomena of interest varied across the 39 studies. The largest proportion of studies (where *n* = number of times each concept was mentioned in the included articles) (*n* = 12, 30.8%) examined gendered experiences of climate change impacts, including vulnerability, adaptation, and coping strategies. Eight studies (20.5%) investigated women's roles in climate‐related decision‐making, governance and policy. Health and wellbeing outcomes were addressed in seven studies (17.9%), while six studies (15.4%) explored women's economic participation, labour, and resource access under environmental stress. Three studies (7.7%) examined cultural and spiritual connections to land and environment, and a further three (7.7%) focused on intersectionality and human rights in climate contexts. Although the studies were categorised by their primary phenomenon of interest, several reported incidental findings that overlapped with other aspects of the extraction framework were identified, which are presented in Appendices B–E. None of the included articles reported on gender diverse groups (including transgender individuals and individuals who identify as gender non‐binary).

#### 4.1.3 Cultural Context (D1)

The included papers reflect the different ways in which women are positioned in their local, dominant societies. To highlight the vastly different socio‐cultural, political, and environmental landscapes that the suite of studies are situated within, the UNDP (2023) Human Development Index (HDI) has been adopted as a proxy indicator to provide a broad, global context. The HDI, which is a composite measure that reflects development in three key dimensions: health (life expectancy), education (mean and expected years of schooling), and standard of living (gross national income per capita).

Table 2 presents the country of each article examined, the number of studies within the country, and the country's Gender Development Index (GDI) and gender‐based Human Development Index (HDI).

**TABLE 2:** Study locations, number of studies and GDIs.

Country	Number of studies	GDI (HDI Female/HDI Male)	HDI female	HDI male
Thailand	2	1.012	0.805	0.796
United States	2	1.001	0.92	0.919
Czech Republic (Czechia)	1	0.989	0.884	0.893
Canada	2	0.988	0.929	0.941
United Kingdom	1	0.987	0.922	0.934
China	2	0.984	0.761	0.773
Australia	2	0.968	0.932	0.963
Zambia	1	0.965	0.554	0.574
Nicaragua	1	0.956	0.648	0.678
South Korea	1	0.944	0.894	0.947
South Africa	2	0.944	0.686	0.737
Uganda	2	0.927	0.505	0.545
Mozambique	1	0.922	0.428	0.46
Ethiopia	1	0.921	0.478	0.519
Sub‐Saharan Africa (unspecified)	2	0.907	0.519	0.572
Bangladesh	1	0.898	0.617	0.688
Mali	1	0.887	0.399	0.45
Iran	10	0.88	0.704	0.8
Benin	1	0.88	0.491	0.558
Nigeria	1	0.863	0.495	0.574
India	3	0.849	0.567	0.668

Further descriptions of each of the articles examined in this review are presented in Table 3.

**TABLE 3:** Study characteristics.

Citation/MMAT Score	Title	Location	Aims	Study design/Method	Population	Intervention/Phenomena of interest	Key findings	Limitations
Allen et al. (2022) MMAT 4	Mothers‚ agency and responsibility in the Australian bushfires: A feminist new materialist account	Australia	To apply a feminist new materialist approach to understand mothers' feelings of responsibility and agency for their children during the bushfires.	*Study design*: Qualitative interviews. *Methods*: mothers were interviewed about their experiences of the bushfires as part of the Mother and Child 2020 study. Data from this study was revisited and analysed using a feminist new materialist approach.	Women who were mothers or became mothers during the Black Summer bushfires. Participants were from Canberra, the Coast of New South Wales during the fires.	Mothers' feelings of responsibility and agency for their children during the bushfires.	Mothers reported feelings of powerlessness and guilt related to their inability to prevent smoke inhalation for their unborn or newborn children.	This study is part of a larger study. This may have impacted recruitment of participants (who might have not been interested in the larger study). Study was small, with a sample of only 25 which may impact applicability to other contexts.
Amondo et al. (2022) MMAT 4	Health gender gap in Uganda: do weather effects and water play a role?	Uganda	To examine how weather variability affects illness among working‐age men and women in Uganda. To measure how water collection time mediates the link between weather anomalies and illness. To identify the sources of the health gender gap, including health‐seeking behaviours. And to measure the health inequalities that could be reduced by equalising resources.	*Study design*: quantitative, observational study with a cross‐sectional and econometric modelling approach. *Methods*: Data from four waves of Uganda's LSMS‐ISA household surveys (2009–2014) was combined with remote sensing weather data.	22,469 working‐age men and women (15–64 years old) in rural Uganda.	Examining how weather variability affects health, both directly and indirectly through water collection time, while exploring gender differences in health outcomes and behaviours.	Low rainfall below the long‐term mean was associated with increased likelihood of illness among both women and men, with a greater effect observed among women; gender‐based health inequalities were estimated to be substantially reduced if resource distribution was equalised.	The lack of data on health adaptation measures, individual‐level weather data, and future climate change scenarios means further research is needed.
Andajani‐Sutjahjo et al. (2015) MMAT 3	Gender and water in Northeast Thailand: Inequalities and women's realities.	North East Thailand—Isaan ethnicity	To explore the gendered roles, responsibilities and inequalities of access to and control over village water resources in a peri‐urban farming community in Northeast Thailand.	*Study design*: The study used a qualitative field research approach. *Methods*: Data was collected through observations, informal dialogues, group interviews and in‐depth interviews conducted in the Isaan dialect, with interviews being tape‐recorded, transcribed, and translated into Thai and English.	Participants were selected using purposive sampling, targeting those involved in water management, including 82 lay‐residents, 34 local leaders and 16 local administrators.	Gendered roles of water collection and the impact of climate change on women.	Gender inequality in water consumption was shaped by household structures and responsibilities. Women were positioned as primary observers and ‘guardians’ of water resources, while male‐dominated, engineering‐focused water management systems failed to adequately recognise women's social capital and community roles.	The study applied limited contextual application.
Md et al. (2022) MMAT 4	Exploring gender and climate change nexus, and empowering women in the South Western coastal region of Bangladesh for adaptation and mitigation.	South Western Coastal Region of Bangladesh (SWCRB)	To identify the vulnerabilities and effects of climate change on women in the South Western Coastal Region of Bangladesh (SWCRB), and to investigate the importance of women empowerment to reduce their inequality and vulnerability in this region.	*Study design*: Mixed Methods *Methods*: The study combined qualitative and quantitative techniques. Qualitative methods included focus groups, case studies, interviews, workshops and field observations. The quantitative component surveyed 320 households and analysed data with cross‐tabulation, chi‐square tests, and symmetric measures using SPSS and Excel.	Inhabitants (men and women) of the Shyamnagar Upazila region in the Southwestern Coastal Region of Bangladesh (SWCRB). Respondents were teachers, community leaders, farmers, non‐governmental organisation representatives, fishermen, farmers, honey collectors and gender practitioners.	Vulnerabilities and effects of climate change on women, and adaptive capacity that fosters resilience.	Women were disproportionately affected by climate change and natural disasters due to intersecting factors including limited access to water and food, distance from cyclone shelters, exclusion from decision‐making, restrictive social conditions, unpaid care responsibilities, poverty and loss of housing.	Some of the quantitative findings were not statistically significant or explored in the discussion/conclusion of the paper.
Asongu et al. (2022) MMAT 5	Women political empowerment and vulnerability to climate change: evidence from 169 countries	Worldwide	Analyse the effect of the political empowerment of women on vulnerability to climate change in 169 countries for the period 1995–2017	*Study design*: Observational, longitudinal study using panel fixed effects regressions; for multi‐site retrospective data from 1995 to 2017. *Methods*: Panel fixed effects regressions is the primary method to analyse the effect of women's political empowerment on vulnerability to climate change. Employs alternative estimation methods such as Tobit, dynamic fixed effects, and generalised method of moments (GMM) to ensure robustness of the findings.	Citizens of 169 countries	The impact of women's social and political empowerment on vulnerability to climate change	Women's political empowerment, including participation in civil society and political processes, was associated with reduced vulnerability to climate change, with effects most pronounced in upper‐middle‐income, Latin American, small and fragile countries, mediated through education, governance effectiveness and public education spending.	The sample size raises data completeness and complexity of cultural context concerns, as well as missed data points. Likewise, the relatively short time frame of data compared to the slow progress of climate change and empowerment could pose limitations to the applicability of results.
Austin et al. (2021) MMAT 5	Drying Climates and Gendered Suffering: Links Between Drought, Food Insecurity and Women's HIV in Less Developed Countries	91 Developing countries	Explore linkages between suffering from drought, food insecurity and women's vulnerability to HIV.	*Study design*: Observational cross‐national study using structural equation modelling (SEM). *Methods*: Data from various sources, including the Food and Agriculture Organisation and the World Bank. SEM is used to model direct and indirect effects of these factors on the percentage of the adult population living with HIV that are women. The study controls for GDP per capita and includes measures of food insecurity and contraceptive use. The variables are time‐ordered to address causality, with independent variables measured before dependent variables. The study reports model fit statistics and provides regression coefficients and standard error estimates.	Women > 15 years in developing countries	Vulnerability to HIV as related to drought and food insecurity.	Drought was found to indirectly exacerbate gender inequalities in the HIV burden through increased food insecurity, with effects mediated by reductions in women's socio‐health status and contraceptive use, resulting in a substantial overall influence on women's share of HIV cases.	Authors note risky sexual behaviours, a key factor in the relationship between food insecurity and HIV, could not be explicitly modelled in their study. The cross‐national approach allows for generalisation across countries, however it cannot fully capture individual behaviours.
Cameron et al. (2021) MMAT 4	Global Crises: Gendered Vulnerabilities of Structural Inequality, Environmental Performance and Modern Slavery	Global ‐ 180 countries	To examine the relationship between environmental stressors associated with climate change, selected structural inequalities and the estimated prevalence of modern slavery cases across 180 countries.	*Study design*: quantitative, correlational study using secondary data analysis. *Method*: Publicly available archival data from records, including the United Nations Development Program (UNDP), the United Nations Office on Drugs and Crime (UNODC), the 2018 Environmental Performance Index (EPI), and the 2018 Global Slavery Index, was used to conduct a regression analysis.	The study population/participants discussed in the paper are women, children and victims of human trafficking from 180 countries.	Number of modern slavery cases.	Indicators of poor environmental health were associated with increased risk of modern slavery among women, with factors such as women's lack of parliamentary representation, suboptimal access to education, tree cover loss, agricultural management practices and air quality contributing to the prediction of vulnerability.	While significant predictors were identified, other potential contributors (e.g., cultural norms, legal frameworks) may have been overlooked. Further, the use of global data might mask regional nuances.
Chapola et al. (2024) MMAT 3	Climate change and its impact on the mental health well‐being of Indigenous women in Western cities, Canada	Western Canada	The study aims to explore the interconnections between climate change and the mental health and well‐being of Indigenous women in Western Canada.	*Study design*: The study used an Indigenist feminist research approach, which combined Indigenist and feminist research methods. *Methods*: The researchers used two Indigenist research methods: learning women's stories and engaging in women's conversations.	The study population/participants are Indigenous women in Western cities in Canada.	Mental health experiences of Indigenous women in Western Canada.	Environmental degradation, combined with ongoing colonial impacts on traditional practices, was associated with adverse mental wellbeing outcomes among Indigenous women.	
Chen and Hansen (2022) MMAT 5	Gender and Power in China's Environmental Turn: A Case Study of Three Women‐Led Initiatives	China	To show examples of what motivates women in China today to take leadership of local environmental projects.	*Study design*: Qualitative, ethnographic fieldwork utilising case studies. *Methods*: Researchers conducted interviews and focus groups with over 100 women in rural and urban areas. The study includes three case studies, each focusing on different locations and organisations. Participant observation was used in two of the cases. Incorporates data from governmental sources and organisational documents, providing a broader context.	Women in leadership of community programs (*n* = 3)	If China's strengthened environmental policies and ideology may help empower women	The elevation of environmental issues such as waste collection and recycling in China expanded opportunities for women's participation and subjectivities in public life; however, these changes did not translate into improved representation or influence at higher levels of political authority, remaining constrained by existing male‐dominated political structures.	The study had a small sample size and limited representation of women across all levels of government. The complex sociopolitical environment may have limited what the participants shared in focus groups/the public sphere.
Das (2024) MMAT 5	Women's experiences and sustainable adaptation: a socio‐ecological study of climate change in the Himalayas	Himalayas	(i) to assess the impact of climate‐induced emergencies on women and (ii) analyse the gendered norms influencing food choices, with the intent of identifying gender‐inclusive measures for climate adaptation and food security	*Study design*: Mixed‐methods cross‐sectional observational study *Methodology*: Multi‐stage random sampling. Qualitative thematic analysis of interviews via NVIVO. Quantitative analysis of surveys via Kappa statistics and Pearson Chi‐square tests.	Households in communities identified as vulnerable	Traditional foods security and climate adaptation	Gender norms were found to exacerbate women's vulnerability to climate change impacts, while women's traditional knowledge played a critical role in supporting sustainable food practices and climate adaptation strategies.	The authors reported restricting the scope of the questionnaire, suggesting a potential limitation in the data collected and thus robustness of results.
Datta et al. (2024) MMAT 5	Decolonizing meanings climate crisis and land‐based adaptions: From Indigenous women's perspectives in Western Canada	Western Canada	Explore Western Canadian Indigenous women's perspectives on land‐based climate adaptations to inform the development of culturally sensitive and gender‐responsive strategies.	*Study design qualitative*—storytelling dialogue and narrative analysis. Collaborative decolonial feminist approach. *Methods*: Thematic coding informed by decolonial feminist frameworks.	Indigenous women from across western Canada (*n* = 15).	Indigenous women's perspectives of climate change	Incorporating Indigenous knowledge systems into climate mitigation and adaptation strategies was identified as essential, with Indigenous women offering perspectives that integrate cultural, gendered and ecological dimensions of climate change impacts.	Authors explain that they prioritised certain narratives, which they explain may have resulted in a limited representation of viewpoints. The effort to decolonize meanings and include Indigenous knowledge may not fully reflect the diverse responses of Indigenous communities to the climate crisis, especially given variations due to geography, culture and socio‐economic factors.
Detraz and Peksen (2017) MMAT 5	In the Aftermath of Earth, Wind and Fire: Natural Disasters and Respect for Women's Rights	148 countries impacted by a significant natural disaster from 1990 to 2011	This paper explores the extent to which natural disasters affect women's economic and political rights in disaster‐hit countries.	*Study design*: Descriptive quantitative *Method*: Utilising the Cingranelli‐Richards (CIRI) dataset (2012) and the Emergency Events Database (EMDAT) to inform the regression analysis.	Women citizens of the included studies	The impact of natural disasters on the rise of systematic gendered discrimination.	Greater disaster severity was associated with reduced government respect for women's economic and political rights, while democratic governance, CEDAW ratification and higher GDP were linked to stronger protection of women's rights, supporting evidence that natural disasters negatively affect women's rights outcomes.	The impact of disasters is likely to be most significant at the subnational level, which is outside of the scope of the study.
Dutta et al. (2024) MMAT 5	Environmental policy implementation, gender and corruption		Explore the relationship between women's participation in politics and the success in enacting environment‐friendly policies. Focus exclusively on whether the negative effect of corruption is strong enough to erode the beneficial environmental policies that women legislators propose and enact at the country level.	*Study design*: Cross‐sectional data analysis of 83 countries *Methods*: Empirical analysis using instrumental variable regressions and matching models.	83 Countries	Gender and corruption in environmental policies	Higher representation of women in parliament was associated with more favourable adoption of climate change policies; however, elevated levels of corruption undermined these benefits, limiting women legislators' capacity to influence environmental policy outcomes.	Study impacted by limited reporting from countries included in the data set.
Eunsoo Kim (2022) MMAT 5	The Effect of Female Personnel on the Voluntary Disclosure of Carbon Emissions Information	South Korea	To ‘examine the impact of female personnel (female executives and female employees)’ on corporate voluntary disclosure policy on carbon emission information.	*Study design*: Quantitative cross‐sectional model research design involving regression analysis *Method*: A cross‐sectional model used to measure accrual quality, with a minimum of 20 firm‐year observations per industry was used. Regression models included ‘year’ and ‘industry fixed effects’.	Non‐financial firms listed on the Korea Stock Exchange (KOSPI and KOSDAQ) from 2014 to 2020.	Corporate voluntary disclosure policy.	Gender equality policies were identified as necessary in South Korea to increase women's representation within corporate governance structures.	This study is limited by its exclusive focus on South Korean companies, which restricts the generalisability of the findings to other national contexts. In addition, the observed associations may be subject to reverse causality, whereby firms with pre‐existing commitments to transparency and sustainability are more likely to promote gender diversity, rather than gender diversity independently driving the observed outcomes.
Floro et al. (2019) MMAT 3	Gender and Perception of Climate Change in Ethiopia	20 villages across 5 regions of the Nile Basin Ethiopia.	To examine the interplay of gender roles, access to information and household dynamics that influence agricultural households' awareness about climate change in rural Ethiopia. Specifically, it investigates the extent to which women's and men's experiences of shocks and differential access to agricultural extension services and farmers' networks explain the difference in their perception of climate change and whether women's voices in household matters can influence the climate perception of the household head.	*Study design*: Descriptive cross‐sectional study. *Methods*: Analysis based on data from a 2004–05 household survey in Ethiopia. The study uses weighted logit and generalised maximum entropy regression methods to examine: (1) gender differences in climate change perception among household heads, (2) effects of exposure to shocks and access to information on awareness and (3) influence of women's voice in households on perception.	1000 rural households in Ethiopia, with 901 male‐headed households and 99 female‐headed households.	The interplay of gender in awareness and understanding of climate change.	Women were less likely than men to perceive climate change risks, due to limited access to agricultural extension services and farmers' networks; however, women's health experiences, access to information, literacy and participation in household decision‐making were associated with increased household awareness and adaptive capacity.	Data was obtained during a larger rural household study. Data may not fully align with the specific objectives of the published study, as it was originally collected for a different purpose.
Ghasemi et al. (2021)	The mediation effect of rural women empowerment between social factors and environment conservation	The Chahar‐mahal and Bakhtiari Province, located in the southwest of Iran	This study examines how empowering rural women mediates the relationship between social factors and environmental conservation, combining empowerment and eco‐feminist theories.	*Study design*: cross‐sectional survey. *Method*: structured interviews using a researcher‐developed questionnaire. Questions were measured on a five‐point Likert scale from strongly disagree to strongly agree and very low to very high.	384 out of 133,158 rural women of the nine townships in this province	Social Factors as predictors for women empowerment in environmental conversations	Women's empowerment in environmental discussions was influenced by family position, spousal behaviour and social support, with increased participation in environmental decision‐making committees and strengthened participatory approaches identified as critical for fostering environmental conservation.	Findings were shaped by the specific geographic and socio‐political context.
Ghosh and Dutta (2024) MMAT 3	Health threats of climate change: from intersectional analysis to justice‐based radicalism	India ‐ The Indian Sundarbans	To understand how climate change combined with social, political and economic factors creates health threats, particularly for marginalised groups such as women and children in the Indian Sundarbans region.	*Study design*: Ethnography *Method*: The study used an intersectional, justice‐based approach with qualitative methods like interviews, focus groups and observation.	Participants included women (16–40), children (10–16), grandparents (45–65), local community members, healthcare providers (formal and informal) and experts in ecology, agriculture and fisheries.	Health impacts of climate change on women	Climate change was found to generate direct health impacts that intersect with existing social, economic and cultural inequalities, disproportionately affecting women and children, while resilience agendas were critiqued for promoting maladaptive, individualised responses through state and private‐sector interventions.	Small study, warranting further research.
Goli et al. (2023) MMAT 5	Are adaptation strategies to climate change gender neutral? Lessons learned from paddy farmers in Northern Iran	Northern Iran	To conduct a gender analysis of the effects of climate change on the adaptation strategies of rice farmers in Mazandaran Province (northern Iran) based on the sustainable livelihood approach.	*Study design*: Qualitative *Methods*: Straussian Grounded Theory (SGT) for analysis. Interviews and focus groups for data collection. Purposeful and theoretical sampling techniques	Female & Male heads of households	Climate change adaptation	Female‐headed households experienced greater vulnerability to climate‐related shocks than male‐headed households, with gendered roles shaping exposure to risk, asset vulnerability and adaptive responses during climate crises.	Small sample size. Limited active participation by some respondents.
(Harnish 2014) MMAT 3	Extractive workload: a mixed‐method approach for investigating the socially differentiated effects of land‐use/land‐cover changes in a southern Zambian frontier	Kulaale region of Zambia	The study aims to investigate the socially differentiated (i.e., gendered and aged) effects of land‐use/land‐cover changes on Gwembe Tonga migrants living in Kulaale, Zambia.	*Study design*: mixed‐methods *Methods*: Integrated existing analyses of remotely sensed imagery, qualitative ethnographic methods including semi structured interviews, with a seasonal resource survey and mapping exercise of a purposive subsample of 20 homesteads across two research zones defined by patterns of agricultural expansion.	Gwembe Tonga migrants living in the Kulaale region of Zambia.	Gender differentiated land use/land cover changes	Deforestation was associated with increased extractive workloads, with boys and men experiencing the highest average workloads across zones and gendered differences indicating that women, men and children experience distinct workload impacts linked to environmental change.	The analysis adopts a resource‐focused perspective rather than an individual‐level approach, with findings shaped by methodological constraints including sample size, scope of resources examined and the use of simplified distance measures.
Hlahla et al. (2022) MMAT 2	Climate‚ Urban Nexus: A study of vulnerable women in urban areas of KwaZulu‐Natal Province, South Africa.	KwaZulu‐Natal, South Africa	(1) Assess if local climate policies in the province incorporate gender considerations and whether these policies are effectively implemented. (2) Investigate how climate variability affects marginalised women and female‐headed households in urban KwaZulu‐Natal, South Africa and their response measures.	*Study design*: Mixed Methods *Methods*: The study collected data via questionnaires, key informant interviews, direct observations and a desktop review of climate change policies. Quantitative data were analysed using descriptive statistics in SPSS, and qualitative data from open‐ended responses and interviews were analysed through inductive thematic analysis.	The questionnaire survey was conducted with 264 households, 81% of which were female‐headed.	Impact of climate change on women in South Africa. Policy	Climate change adversely affected the livelihoods of marginalised women in urban KwaZulu‐Natal, South Africa, with limited knowledge and resources constraining adaptive capacity and resulting in predominantly reactive coping strategies, particularly among impoverished female‐headed households.	Study uses convenience sampling which may have introduced selection bias. Overrepresentation of female‐headed households may skew data. Unclear to what extent the impacts effect all people (as opposed to just women).
Imran and Ozcatalbas (2020) MMAT 3	Determinants of household cooking fuels and their impact on women's health in rural Pakistan	Rural Pakistan	To investigate the energy consumption patterns of rural households, analyse the factors affecting the household choice of cooking fuels, and discuss the burden of the use of cooking fuels on rural women.	*Study design*: cross‐sectional, quantitative descriptive design. *Methods*: Data was collected using a structured questionnaire administered to a random sample of 196 households, with the household head as the unit of analysis.	196 rural households selected from 4 districts (Muzaffargarh, Faisalabad, Khushab and Rawalpindi) in the Punjab province of Pakistan, using a multistage random sampling approach.	Factors influencing the choice of cooking fuels.	Communities in rural Pakistan, predominantly rely on biomass fuels for cooking, which was associated with adverse health outcomes for women, including respiratory symptoms, headaches, and eye problems, while use of improved cookstoves and modern fuels was linked to reduced health impacts and influenced by household socioeconomic and infrastructural factors.	The study design limited the researcher's ability to evaluate various other important health indicators that may have influenced the results. Health data was not stratified, meaning that the health claims are non‐conclusive. Small sample size.
Jannat and Kusakabe (2024) MMAT 5	Women's Participation in Community‐Based Adaptation to Climate Change (CBACC): An Intersectional Analysis	Bangladesh	Explore which women participate in community‐based adaptation (CBA) from an intersectional perspective.	*Study design*: Qualitative *Methods*: In‐depth interviews (IDIs) with women from different ethnic and religious groups	Women involved in community climate change adaptation groups.	Women's participation in climate change adaption.	Participatory approaches implemented by NGOs were found to inadvertently exacerbate inequalities among women, with intersectional barriers to participation shaped by insecurity, family support, literacy levels and administrative biases within community‐based activities.	Narrow population sample.
Liddell et al. (2024) MMAT 5	Environmental Reproductive Justice, Indigenous Health Knowledge and Indigenous Women on the Climate Frontlines	United States ‐ Gulf Region	Explore the overlooked ways that place interacts with Indigenous women's cultural reproductive labouring by examining the ways that traditional health and healing knowledges and practices are being threatened by climate change‐induced environmental degradation.	*Design* Qualitative *Methods*: Semi‐structured interviews with 31 self‐identified women members of a U.S. Gulf South tribe.	Indigenous Women in the United States	Cultural knowledge/legacy in the context of climate change	Environmental change and displacement were associated with concerns about future knowledge transmission, with significant disruption to the sociocultural reproduction of Indigenous health and healing practices due to environmental degradation and the increasing dominance of Western medical knowledge.	Narrow context and small sample size.
Monk et al. (2019) MMAT 4	Gendering pacification: policing women at anti‐fracking protests	The United Kingdom	To understand how women protesters at the Barton Moss anti‐fracking protests were policed differently and more violently compared to men and how this gendered policing was used to enforce compliance and send a message that protest is illegitimate and dangerous.	*Study design*: qualitative, longitudinal case study approach. *Methods*: semi‐structured interviews.	27 protesters (16 men, 11 women) at the Barton Moss Community Protection Camp.	Gendered policing of protestors	Women protesters at the Barton Moss Community Protection Camp experienced gendered policing practices, including sexual harassment and violence, indicating that gendered state violence may function as a mechanism of social control to deter women's participation in protest.	Small study sample applicable to one context.
Munala et al. (2023) MMAT5	Climate Change, extreme weather and intimate partner violence in East African Agrarian‐Based economies	Uganda, Zimbabwe and Mozambique	To explore the relationship between extreme weather events, specifically severe floods and droughts, and intimate partner violence in agrarian‐based economies in East Africa, focusing on Uganda, Zimbabwe and Mozambique.	*Design*: An ecological study design examining the relationships between exposure and outcome variables at the group or population level. *Methods*: Utilised secondary data. IPUMS‐DHS provided household survey data on intimate partner violence. EM‐DAT provided data on weather‐related disasters.	Women aged 15–49 who had been married or lived with a partner and who reported on their experiences of intimate partner violence in the past year. The sample sizes were 1739 women in Uganda, 5280 in Zimbabwe and 5824 in Mozambique.	Extreme weather and intimate partner violence	Severe weather events, particularly floods and droughts, were associated with increased reports of intimate partner violence in agricultural communities in Uganda, Zimbabwe and Mozambique.	The lack of precise location data for weather events could lead to some misclassification, but the authors argue this would not significantly impact the overall findings. The authors used weather data that affected a large number of people to help address potential misclassification, and their findings are consistent with previous research on the connections between climate factors and intimate partner violence.
Obossou et al. (2023) MMAT 3	Gender dimensions in the adoption of climate‚ smart agriculture technologies in response to climate change extremes in Benin	Benin, sub‐Saharah Africa	To investigate the gender dimensions in the adoption of climate‐smart agriculture (CSA) technologies among smallholder farmers in Benin.	*Study design*: Mixed methods cross‐sectional design. *Method*: The study design and methods were a mixed‐methods approach, using both quantitative structured interviews and qualitative focus group discussions and key informant interviews.	Small‐scale farmers in northern Benin.	The uptake of Climate Smart Agriculture technologies	Gender differences were observed in the adoption of climate‐smart agriculture technologies, with men adopting more practices overall and women favouring less labour‐intensive options, reflecting disparities in access to land, resources, information, and decision‐making power.	Sample size may not fully capture the diversity of small‐scale farmers in Northern Benin, limiting its representativeness
(Okere et al. 2024) MMAT 4	Pollution, governance, and women's work: Examining African female labour force participation in the face of environmental pollution and governance quality puzzles.	Sub‐saharan Africa	To examine the impacts of governance quality and environmental pollution on gender economic outcomes, specifically female labour force participation, in Sub‐Saharan Africa.	*Study design*: Quantitative descriptive design with a dynamic panel threshold model. *Methods*: The study design and methods involve using the dynamic panel threshold model to examine the impacts of governance quality and environmental pollution on female labour force participation in Sub‐Saharan Africa.	28 Sub‐Saharan African (SSA) countries over the period 1996 to 2020.	Gender economic outcomes arising from environmental pollution and governance.	Higher ecological footprints were associated with reduced female economic participation in Sub‐Saharan Africa, while sustainable development and improved governance were identified as critical factors for enhancing women's economic involvement.	The study relies on secondary data from global indices. Different countries may use varied methods or standards for collecting data included in global indices (e.g., ecological footprint or governance quality). This inconsistency may have introduced bias or error, reducing the comparability of data across countries.
Ojakorotu and Olajide (2018) MMAT 3	Gendering climate change and human rights in Africa: the case of coastal communities in Lagos state Nigeria.	Lagos state Nigeria	The study aims to examine the human rights impacts of climate change on women in selected coastal communities in Lagos State, Nigeria, using the feminist political ecology framework.	*Study design*: Qualitative study design *Methods*: Interviews were conducted with a total of 10 respondents, 5 each from the coastal communities of Ilaje and Makoko in Lagos State, Nigeria. Respondents were selected using stratified random sampling. Responses were analysed using the Atlas software.	Residents of the coastal communities of Ilaje in Shomolu Local Government and Makoko in Lagos Mainland Local Government in Lagos State, Nigeria. A total of 10 respondents were interviewed, 5 from each community.	Intersection of climate change with women's human rights	Climate change‐related flooding in coastal communities in Lagos, Nigeria, was associated with violations of women's economic, social, cultural and political rights, exacerbated by inadequate infrastructure and limited institutional support.	Small sample size limited to one context. Some generalisations about women's rights made throughout. May have introduced selection and confirmation bias that privilege women's experience.
Patrick and Capetola (2011) MMAT 2	It's here! Are we ready? Five case studies of health promotion practices that address climate change from within Victorian health care settings	Victoria Australia	To explore and describe examples of health promotion practices addressing climate change and sustainability in Victorian health care settings through a case study approach.	*Study design*: Qualitative research using a case study design. *Methods*: Data was collected through individual and group interviews with 10 health care practitioners in 5 Victorian health care settings, supplemented by documentary evidence.	10 female health care practitioners (age range 20–60 years) from 5 Victorian health care agencies that had explicitly identified climate change or environmental sustainability as a service or program priority.	Health promotion practices addressing climate change and sustainability	One case study, on women's health intervention, highlighted the role of advocacy, research and health promotion frameworks, underpinned by social justice, systems thinking and interdisciplinary approaches, in responding to climate change‐related health challenges.	One case study. Non‐specific details of intervention, research areas and outcomes.
Pinho‐Gomes and Woodward (2024) MMAT 2	The association between gender equality and climate adaptation across the globe	Global	To investigate the association between gender inequality and vulnerability and readiness to adapt to climate change across the globe.	*Study design*: ecological, observational, cross‐sectional design. *Methods*: simple linear regression were used to investigate the association between country‐level measures of climate adaptation (ND‐GAIN index) and gender equality (GGGI and GII indices) using data from 2022, with scatterplots and loess curves to explore the linearity of the associations.	146 countries from around the world, with a focus on analysing country‐level data on gender equality and climate adaptation.	Gender inequality and vulnerability/readiness to adapt to climate change.	Higher levels of gender equality were associated with greater national capacity for climate change adaptation, while gender inequality was linked to increased vulnerability and reduced adaptive readiness.	The study draws conclusions based on group‐level data (countries) that may not accurately apply to individuals or subgroups within those countries. The design only provides a snapshot in time, so it cannot establish causality or determine how relationships between gender equality and climate adaptation evolve over time.
Puwajarernpong et al. (2023) MMAT 5	Gender and disaster management: A case study of Prik Tai community, Songkhla Province, Thailand	Songkhla Province, Thailand	To examine community‐based disaster (flood) management processes and explore gender roles in disaster management. To understand how the roles and responsibilities of Thai women in production, reproductive work and community work can be recognised, valued, and incorporated into policy and planning, especially in disaster management, to alleviate their excessive workload and promote social and gender equity.	*Study design*: Qualitative. Case Study *Methods*: qualitative investigation using participant observation, non‐participant observation and semi‐structured interviews to examine gender roles in disaster management and everyday life within the Prik Tai community.	The study included key informants (18 main flood management informants from 9 households with both men and women, 3 community leader households, 3 community member households, and 3 vulnerable households), casual informants (4 community leaders) and general informants (26 people involved in disaster management).	Experiences of women during disaster management	Women in the Prik Tai community played central roles in disaster management at household and community levels through care and community work; however, this labour was largely undervalued and unrecognised, contributing to excessive workloads and increased health risks amid climate change and demographic shifts in Thailand.	The findings are specific to the Prik Tai community and may not be applicable to other communities in Thailand. Although the study acknowledges men's contributions, it primarily focuses on women's roles, which might overlook the full spectrum of gender dynamics in disaster management.
Saville and Adams (2021) MMAT 4	Environmental justice in the American south: an analysis of black women farmworkers in Apopka, Florida	Apopka, Florida	The overarching goal of this research was to examine how intersectional oppressions influence environmental injustices by reviewing Apopka to ask: (a) how the policies and socio‐historical context of the agricultural industry in the American South contributed to injustices experienced by Black women farmworkers in Apopka, Florida; and (b) how race, gender, and class intersected and intersect to create and legitimise environmental injustices in the workplace for these farmworkers	*Study design*: Qualitative *Methods*: semi‐structured in‐depth interviews, (*n* = 33) newspaper and media coverage, and archival materials.	Initial interviews were conducted with 15 Black women farmworkers in Apopka. Further interviews were carried out with former farmworkers, their Black women family members, politicians, medical professionals, environmentalists and activists. These additional interviews included three Black women who were family members of former farmworkers, two Black women and one Latina woman who were community activists, four white men and one Black man who were politicians, three white men and one white woman who were medical professionals and two white men and one white woman who were environmentalists.	Experiences with agricultural labour, perceptions of injustices in the farmworker community, and perceptions about environmental justice issues such as occupational exposures and environmental illnesses	The muck farms in Apopka, Florida, exemplified environmental injustice shaped by intersecting race, class and gender inequities, where Black women farmworkers experienced hazardous working conditions, pesticide exposure, low wages and inadequate care, with limited attention given to long‐term health impacts despite environmental remediation efforts.	Some of their interviews were not finished as farmworkers passed away, introducing attrition bias.
Segnestam (2017) MMAT 5	Gendered Experiences of Adaptation to Drought: Patterns of Change in El Sauce, Nicaragua	Nicaragua	Interprets gender differentiations in vulnerability in the interplay between structures and resources.	*Study design*: Qualitative *Methods*: Semi structured interviews with 47 participants (9 male heads of household, 9 women in male‐headed households, 8 female heads of household) conducted over three occasions.	Female and Male heads of households.	Vulnerability, coping and adaptation to drought.	Gender differences were found to shape vulnerability, coping and adaptation to drought, with women's limited access to cultural and other forms of capital constraining their capacity to mitigate livelihood impacts within unequal social hierarchies.	Narrow context (findings not broadly applicable).
Shayegh and Dasgupta (2024) MMAT 4	Climate change, labour availability and the future of gender inequality in South Africa	South Africa	To investigate the impact of climate change on gender inequality in the labour market in South Africa.	*Study design*: Longitudinal panel study. *Methods*: A longitudinal panel study used data from South Africa's National Income Dynamics Study (NIDS) survey (2008–2017). Econometric analysis with a truncated Poisson model assessed the impact of weekly maximum temperature on labour supply for workers.	The study population/participants are the households and individuals surveyed in the National Income Dynamics Study (NIDS), a nationally representative longitudinal survey of households in South Africa.	Labour availability and correlations with extreme weather patterns.	Climate change was associated with reduced availability of low‐skilled female labour in highly exposed sectors, leading to relative wage increases for women and narrowing gender wage gaps, although overall economic output declined and outweighed these distributional gains.	Jobs are divided into just two categories: high‐exposure and low‐exposure to heat. Study also assumes clear distinctions between high‐skilled and low‐skilled workers, but real‐life skill levels and job types vary more. Finally major policy change and effects of child labour are not factored into the model.
Shuaib et al. (2019) MMAT 5	Gender Ideologies and Climate Risk: How is the Connection Linked to Sustainability in an African City	Kampala, Uganda	To examine how gender ideologies intersect with climate risks and how this connection links to sustainability in an African city.	*Study design*: Qualitative case study *Methods*: Three focus group discussions (women‐only, men‐only, mixed‐sex) of eight residents per group. Key informant interviews with 45 participants from local community environmental organisations. Review of secondary data, including a UNHABITAT report	Residents of Kasubi‐Kawaala, an unplanned settlement in north‐western Kampala. 20 men and 25 women involved in environmental organisations.	The intersection between gender ideologies and climate risks such as flooding, sanitation crises, indoor air pollution and urban transport challenges	Conventional gender ideologies were found to intensify women's unpaid care responsibilities and restrict mobility and economic participation ‐increasing vulnerability to climate‐related risks.	Small and localised sample. Potential selection bias of participants.
(Soku et al. 2023) MMAT 3	Environmental tax, carbon emissions and female economic inclusion.	64 countries globally	(1) To assess the impact of carbon emissions on the level of female employment/participation in the economy. (2) To examine whether the imposition of environmental tax increases or decreases the level of female employment in an economy. (3) To examine the joint impact of carbon emissions and environmental tax on female economic inclusion.	*Study design*: quantitative, cross‐sectional, econometric design. *Methods*: The study uses macro‐level, country‐level data from the World Bank and OECD, covering 64 countries over the period 1994–2020.	Data from 64 countries.	Environmental tax and female economic inclusion	Environmental taxation was identified as a potentially effective mechanism for reducing carbon emissions and supporting sustainable development, contingent on equitable and progressive design that promotes female economic inclusion and avoids disproportionate burdens on vulnerable groups.	A key finding was that green taxes increase labour participation for women. This analysis may be subject to confounding, as jurisdictions with green tax policies may already exhibit more inclusive labour markets, stronger education systems and supportive gender policies that independently influence women's employment outcomes.
Sommer et al. (2022) MMAT 3	Defend women's rights and save the trees: a cross‐national analysis of women's immovable property rights and forest loss	Global	To examine the association between women's legal rights to immovable property (such as land and infrastructure) and forest loss in low‐ and middle‐income countries.	*Study design*: cross‐sectional analysis. *Methods*: using ordinary least squares regression and two‐stage instrumental variable regression to examine the association between women's immovable property rights in 2000 and forest loss from 2001 to 2015 across 65 low‐ and middle‐income countries.	65 low‐ and middle‐income countries, for which data on women's immovable property rights in 2000 and forest loss from 2001 to 2015 were analysed.	Property rights and forest loss	Stronger property rights for women were associated with lower rates of forest loss in low‐ and middle‐income countries, while no significant relationships were observed between forest loss and democratic governance or economic indicators.	The cross‐sectional nature of the data prevents establishing causality between women's property rights and forest loss. It is therefore unable to directly test how women's property rights affect forest loss and suggests that future qualitative research could better elucidate these mechanisms.
Vésier and Urban (2023) MMAT 4	Gender inequalities in heat‐related mortality in the Czech Republic	Czech Republic	To quantify heat‐related mortality and identify potential sex and gender inequalities in heat vulnerability in the Czech Republic.	*Study design*: quantitative, ecological, observational and time‐series design. *Methods*: utilises a quasi‐Poisson regression model with a distributed lag non‐linear model (DLNM) to analyse the association between daily temperature and mortality in the Czech Republic from 1995 to 2019.	Participants were the entire population of the Czech Republic, with 1,079,302 deaths recorded during the summer months.	Heat and mortality	Women experienced higher mortality risk from extreme heat than men, particularly among those aged over 85 years, with divorced women identified as especially vulnerable, underscoring the importance of incorporating sex and gender considerations into heat adaptation strategies.	The analysis was constrained by limited demographic variables, with the absence of broader socioeconomic indicators restricting deeper examination of differential vulnerability to heat‐related outcomes.
Wood et al. (2021) MMAT 5	Decision‐making capacity to address climate‐induced food insecurity within women‐led groups in Southern Mali.	Southern Mali	To explore how gender and age impact women's perceptions of and responses to climate change‐induced food insecurity in Southern Mali.	*Study design*: qualitative, interviews. *Methods*: used a qualitative interview approach, conducting 41 semi‐structured interviews with participants from 6 villages in the Koutiala Cercle region of Mali.	The study population/participants are subsistence farmers in the Koutiala Cercle region of Southern Mali, belonging to the Bambara ethnic group.	Perceptions and response to climate change.	Generational differences in perceptions of climate impacts were evident, with elder women recalling greater past food and rainfall availability and young women reporting current scarcity, while gendered power dynamics within women‐led agricultural collectives and households shaped decision‐making and labour responsibilities.	The analysis prioritised household‐level dynamics rather than individual women's experiences, limiting insight into intra‐household and intra‐group power relations that may shape food insecurity and related outcomes.

### 4.2 Research Question 1

Which structural and systemic factors impacting gender equity have been identified?

#### 4.2.1 Systemic Oppression and Power Dynamics (D2)

The literature consistently highlighted a convergence of structural and social forces that sustain and reinforce the oppression of women (Figure 3). Systemic oppression and power dynamics that impact women's resilience to endure environmental threats were reported in 76% of the retained articles. Common codes elucidated in the literature included financial exclusion and lack of access to material resources (*n* = 19), underrepresentation in governance and decision‐making (*n* = 14), prescriptive gender norms (*n* = 11), participation in and expectations of domestic and market labour (*n* = 12), education access and lack of access to information (*n* = 8), underlying health issues (*n* = 5), restricted mobility (*n* = 4), rural living and isolation (*n* = 4), motherhood (*n* = 4) and colonialism (*n* = 2) (Appendix B).

As an example, access to education was a challenge for many women (Hlahla et al. 2022), which in some instances perpetuated barriers to harnessing economic opportunities (Cameron et al. 2021), reinforcing systemic limitations on their personal freedoms and capacity to adapt and respond to climate change (Floro et al. 2019; Hlahla et al. 2022; Imran and Ozcatalbas 2020; Jannat and Kusakabe 2024; Pinho‐Gomes and Woodward 2024) (Appendix B).

#### 4.2.2 Differing, Layered Impacts of Environmental Crises on Women (D3)

Extending from structural factors previously described, articles reported how gendered social and economic inequalities intersect with environmental threats to produce distinct and sometimes disproportionate impacts for women (Figure 4). The impacts of environmental threats on women were reported in 61% of articles (Appendix C) and included health impacts (*n* = 16), financial impacts (*n* = 13), safety and security (*n* = 10), impacts on women as mothers and carers (*n* = 8), and impacts on holistic systems of wellbeing (*n* = 4), which encompass relational values and connection to culture, traditions and land, which can be diminished by the loss of species, practices or access to significant places.

**FIGURE 7:**
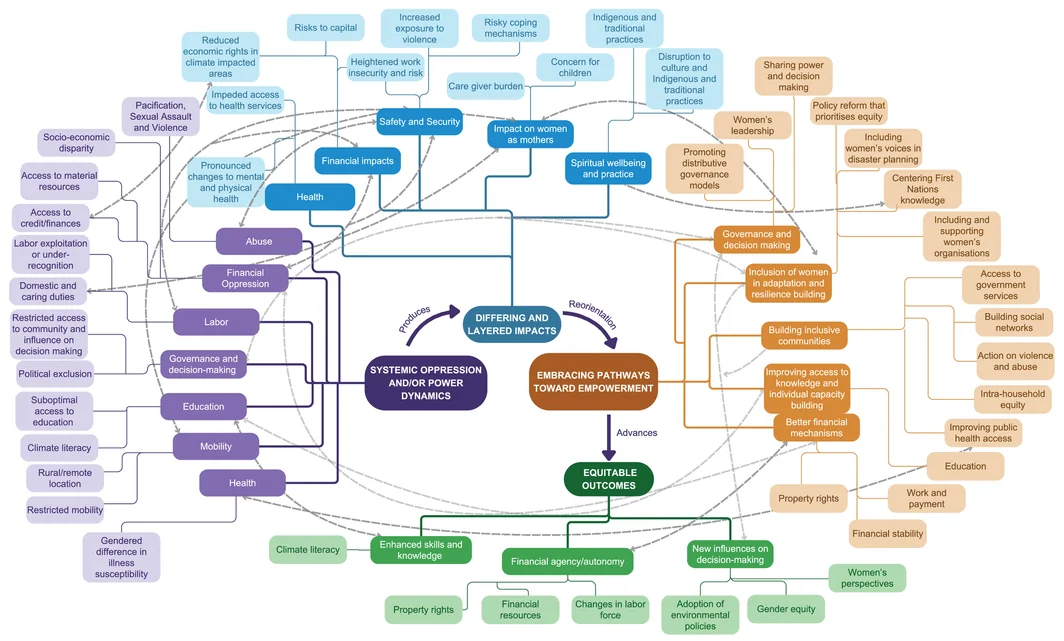
Links between key findings in the literature.

Health impacts identified in the literature included increasing mental health issues including anxiety and stress; illnesses from contaminated water (particularly during floods); malnutrition from food insecurity; fatigue; gynaecological disease and sexually transmitted diseases; heat stress; physical injury and sleeplessness (Chapola et al. 2024; Ghosh and Dutta 2024; Goli et al. 2023; Hlahla et al. 2022; Monk et al. 2019; Ojakorotu and Olajide 2018; Vésier and Urban 2023).

Impacts on spiritual wellbeing were also identified, particularly among Indigenous women (Liddell et al. 2024). Environmental degradation is disrupting cultural practices, sacred rituals and connection to Country (Chapola et al. 2024), undermining women's spiritual resilience and cultural continuity (Chapola et al. 2024). This too presents considerable health risks, deeply affecting Indigenous social and emotional wellbeing as an integral aspect of overall health. Additionally, access to the health system was further constrained during extreme weather events, perpetuating further challenges (Shuaib et al. 2019).

### 4.3 Research Question 2

Which pathways to empowerment, agency and redistribution of power for women and gender‐diverse populations have been identified?

#### 4.3.1 Pathways Toward Gender Justice (D4)

Pathways toward greater gender justice were reported in 64% of the retained articles (64%) (Appendix D). Factors that were grouped as a pathway included gender‐equitable governance and decision making (*n* = 18); representation of women in adaptation and resilience activities and planning (*n* = 16); building inclusive communities (*n* = 13); improving access to education as well as individual capacity building more generally (*n* = 10); and increasing women's financial freedom, agency and autonomy (*n* = 9) were all pathways that may promote a redistribution of power and agency toward greater gender equity (*n* = 25) (Appendix D).

Many studies emphasised the importance of building inclusive communities as a means of advancing equality and gender justice. Key aspects included improving women's access to government services (Floro et al. 2019), particularly social protection schemes, which are vital during climate‐related disruptions (Ghasemi et al. 2021). The literature also pointed to the value of strengthening women's social networks (Obossou et al. 2023) through sustained investment in community groups (Ghasemi et al. 2021), communities of practice (Hlahla et al. 2022), and women‐led organisations (Chen and Hansen 2022). Actions to reduce violence against women (Munala et al. 2023) and shift power dynamics within families (Allen et al. 2022; Ghasemi et al. 2021), thereby enhancing women's decision‐making agency and social status, were identified as important. Reducing the caregiving burden placed on women was also linked to improved climate resilience (Allen et al. 2022).

Improving women's participation in governance and decision making was also highlighted as being critical. Strategies that promoted changes to governance and decision‐making included: distributive governance, which encompassed gender inclusive (Allen et al. 2022) multi‐level governance (Ojakorotu and Olajide 2018); women's leadership (Chapola et al. 2024; Chen and Hansen 2022; Goli et al. 2023; Hlahla et al. 2022) sharing power and decision making (Ghasemi et al. 2021; Pinho‐Gomes and Woodward 2024; Wood et al. 2021) and equality in policy‐making (Detraz and Peksen 2017; Hlahla et al. 2022; Jannat and Kusakabe 2024; Ojakorotu and Olajide 2018).

### 4.4 Research Question 3

What outcomes have been reported from efforts to advance gender equity in the context of the Triple Planetary Crisis?

#### 4.4.1 Outcomes of Gender Justice (D5)

Outcomes of improved gender justice were reported in 38% of the articles (Appendix E). Key outcomes that were reported related to women's leadership (*n* = 8); the promotion of women's financial agency (*n* = 7), and improved education (*n* = 2). The literature identified that investing in women's participation in governance can contribute to the development of more climate‐considerate policies (Asongu et al. 2022; Dutta et al. 2024; Eunsoo Kim 2022; Goli et al. 2023). Progress toward gender equity through enhanced leadership opportunities for women was also seen to support the creation of more resilient and adaptive systems (Asongu et al. 2022) and was associated with reduced societal vulnerability to climate‐related impacts (Asongu et al. 2022; Das 2024).

### 4.5 Research Question 4

How are nurses and other health professionals, women and gender‐diverse individuals represented in this body of literature?

#### 4.5.1 Health Professionals (D6)

No articles reported on the gendered experiences of healthcare professionals in the Triple Planetary Crisis. Three articles explored the health system dimensions of the Triple Planetary Crisis, highlighting that health needs are shifting (see above domain on layered impacts), requiring new responses from health workers (Ghosh and Dutta 2024; Patrick and Capetola 2011).

## 5 Discussion

This scoping review sought to identify and report how gender justice is conceptualised and discussed in contemporary health literature in relation to the Triple Planetary Crisis, with a particular focus on the experiences of women and gender‐diverse populations and the representation of nurses and other healthcare professionals dominated by women. This exposure is mediated by intersecting structural and social factors which compound existing forms of disadvantage and produce layered and mutually amplifying vulnerabilities. Across the reviewed studies, gender justice is consistently identified as a critical pathway for improving outcomes for women. Notably, while gender justice was consistently positioned as a pathway to improved outcomes, the reviewed studies operationalise the concept in diverse ways, emphasising different structural, social and relational dimensions, which contributes to heterogeneity in the conceptualisation of gendered impacts and pathways. In some cases, these pathways were associated with more equitable and effective outcomes. These findings are elaborated in the sections below and synthesised visually in Figure 7.

### 5.1 Structural Barriers—Women's Access to Education and Economic Rights

A key finding from this review was that structural barriers are shaping women's access to opportunities, impacting their adaptive capacity in the face of the Triple Planetary Crisis. Financial barriers, identified in almost half the retained articles, were a critical factor in shaping how women respond to the Triple Planetary Crisis. There were examples of women, particularly young women, not having equitable access to finances, including access to their own personal finances as well as their ability to take out loans or credit (Chapola et al. 2024; Floro et al. 2019; Obossou et al. 2023). Although the literature did not establish a causal link, several articles reported that limited financial autonomy was a factor that exacerbated women's marginalisation, thereby increasing their vulnerability to exposure. For example, a qualitative study conducted on women's perceptions of and responses to climate change‐induced food insecurity in Southern Mali identified that ‘young women [were] not granted the power to use their earnings to respond to their households' food insecurity’ (Wood et al. 2021).

Financial barriers not only limit women's capacity to respond to the Triple Planetary Crisis but are also aggravated by the crisis, reinforcing and widening pre‐existing social and economic disparities. For example, Detraz and Peksen (2017) found that during severe disasters, women's economic rights were much less likely to be protected or valued. They contextualised their findings with the example after Hurricane Katrina, when the Bush administration suspended minimum wage laws in affected areas, a move that disproportionately disadvantaged women concentrated in low‐paid work (Detraz and Peksen 2017).

In many instances, these structural barriers culminated in heightened susceptibility to adverse health outcomes from environmental shocks and stressors. The literature highlighted that health impacts spanned from physical illness and injury, heat stress and malnutrition, to mental health issues such as anxiety and stress (Chapola et al. 2024; Ghosh and Dutta 2024; Goli et al. 2023; Hlahla et al. 2022; Monk et al. 2019; Ojakorotu and Olajide 2018; Vésier and Urban 2023). This pattern is consistent with the broader literature, which similarly documents that climate‐related disasters exacerbate both physical and mental health burdens, while deepening pre‐existing health inequities for women (Keenan et al. 2024; Stone et al. 2022).

### 5.2 Gendered Norms—Care Giving

Beyond structural disadvantages, women experience the Triple Planetary Crisis through the added emotional and practical burden of caregiving, shaped by cultural and societal expectations that position them as primary carers responsible for the needs of their children and families. By and large, the literature highlighted how entrenched gendered roles and social norms are impacting how women live and work (Mari‐Dell'Olmo et al. 2022; Saville and Adams 2021; Shuaib et al. 2019). In several contexts, women were socialised to absorb primary caretaking responsibilities for children and the elderly (Allen et al. 2022; Ghosh and Dutta 2024; Puwajarernpong et al. 2023; Shuaib et al. 2019). This finding is unsurprising in the context of the broader literature, which frequently highlights that women's caregiving roles are often socially and contextually prescribed, with their own needs often conflated with those of children and the elderly (Langer et al. 2015).

This review also identified that women reported feeling a strong sense of responsibility for protecting children from environmentally‐linked harms such as food insecurity (Ghosh and Dutta 2024) and poor air quality (Allen et al. 2022). This can place an undue emotional burden on women who often absorb these responsibilities, causing them to experience heightened anxiety, guilt and emotional distress (Allen et al. 2022; Ghosh and Dutta 2024). For example, Allen et al. (2022) outlined how the public health messaging to ‘stay inside’ to minimise smoke inhalation during the Australian Black Summer bushfires resulted in distress for some mothers. These women reported experiencing intense guilt either for having to take their children out on errands or, conversely, for restricting them from playing outdoors (Allen et al. 2022).

### 5.3 Women's Work

Limited recognition of domestic work and restricted access to decision‐making opportunities are key concepts for interrogation, to advance gender justice (Global Health 50/50, 2024). Not only do gendered norms drive women's caring responsibilities, they also continue to impact women's working conditions as well as opportunities for leadership and influence on decision‐making. Women's work in both domestic and labour markets continues to be reported as being underrecognized (Goli et al. 2023), unpaid (Obossou et al. 2023), in some instances exploited (Cameron et al. 2021), and a key barrier to inclusion in community and political decision‐making (Andajani‐Sutjahjo et al. 2015; Das 2024).

Environmental shocks and stressors were found to exacerbate these challenges: impeding women's ability to work and generate income (Ojakorotu and Olajide 2018). In some instances, the shocks and stressors of extreme weather compelled women to take on additional labour, often under unsafe or exploitative conditions where protections were weakened or absent (Goli et al. 2023). The literature thus reflects that uniform, ill‐informed neo‐liberal responses to the Triple Planetary Crisis may not be equitably accessible or practical across diverse socioeconomic and cultural contexts (Obossou et al. 2023). Instead, recognising, reducing and compensating women's labour (Puwajarernpong et al. 2023; Shayegh and Dasgupta 2024) and addressing financial instability and/or poverty (Goli et al. 2023; Puwajarernpong et al. 2023; Wood et al. 2021) are critical factors for effective policymaking in the context of the Triple Planetary Crisis.

### 5.4 Displacement

In addition to financial impacts, women are increasingly facing displacement arising from the Triple Planetary Crisis. The number of people internally displaced by climate‐related disasters such as cyclones, storms, wildfires, droughts and floods is increasing each year. In 2024, 45.8 million people were internally displaced by climate disasters, with 29 countries reporting their highest number of internal displacements to date (IDMC 2025). Each of the studies examined in this review was from countries that reported internal displacements as a result of climate‐related disasters (IDMC 2025), emphasising the compounding and eroding impacts of ongoing climate change.

Case studies from across the world describe how expanded insecurities associated with displacement are creating multi‐layered sites of vulnerability for women, girls and gender‐diverse individuals (Ayeb‐Karlsson 2020; Oyilieze et al. 2022; Schuster et al. 2024; Singh‐Peterson et al. 2025). The IDMC (2025) report that across countries, internally displaced households with higher levels of vulnerability were most often headed by women or included people with disabilities. Other studies report how women are most likely to be displaced following extreme weather events, citing the 2022 displacement of 8.2 million people in Pakistan following extensive flooding events, of which 80% were women (UHRO 2022).

These impacts are multi‐faceted and nuanced, anchored in local socio‐cultural contexts and socio‐economic realities, yet at a global scale the movement of people escaping from untenable living situations is rapidly increasing. The other significant cause of internal displacement is conflict, which can follow in the aftermath of climate‐related disasters. In slow‐onset climate events, competition for water, fuel and food can intensify, exacerbating existing precarity (IDMC 2024). Accordingly, future‐focused resilience and adaptation planning needs to consider the very real prospect of ongoing and concurrent sites of displacement and potential for conflict should adaptation efforts fail.

### 5.5 Cultural Context

Because socio‐cultural contexts are diverse and locally nuanced, the findings of this review cannot reasonably be extrapolated and applied evenly across different settings. The social, economic and political conditions that shape women's lives, including their rights and freedoms, manifest differently across countries and cultures. This review drew on the Human Development Index (HDI) and the Gender Development Index (GDI) to provide a broad indicator of these variances in health, education and living standards between women and men across countries. For instance, two studies were analysed from Thailand, which has a GDI greater than one, indicating that women have higher living standards, higher levels of education and longer lives than men. In contrast, three studies were located in India, where women are disadvantaged in the HDI in comparison to men (GDI of 0.849). This indicator is intended to reflect biased social norms which produce gendered patterns of land ownership, where only 13% of rural women in India own land, although 85% of rural women are actively engaged in agricultural work (UN Women 2022).

Other studies were located in countries like Mozambique and Mali, which are greatly impacted by compounding extreme weather events and civil conflicts. In Mozambique, for example, up to 1.3 million people have been internally displaced since 2020. Up to 25% of the 580,000 internally displaced people in 2024 reported feeling fearful of gender‐based and sexual violence, whilst 25% of those displaced reported having insufficient water for basic needs (IDMC 2024). These sobering statistics portray the vastly different and complex contexts described in the included studies. As such, while this scoping review has identified familiar themes and topics across diverse, socio‐political, economic, environmental and cultural contexts, it does not attempt to extrapolate and globalise key trends in the literature.

### 5.6 Progressing Toward Gender Justice

Addressing the growing impacts of the Triple Planetary Crisis requires solutions that embrace gender justice. What is required is not a new or separate agenda to advancing gender equity, but a renewed commitment to implementing the existing one with urgency and within the context of the Triple Planetary Crisis. Many of these solutions require further amplification.

As a critical first step toward gender justice, women must be better represented in leadership and governance in responding to the Triple Planetary Crisis. For example, negotiations at the Climate Change Conference of the Parties must be decolonised and prioritise the lived experiences of women (van Daalen et al. 2024). Women's integration in natural disaster planning (Puwajarernpong et al. 2023), investing in women‐led organisations and social networks (Chen and Hansen 2022; Goli et al. 2023; Puwajarernpong et al. 2023), and respecting and integrating First Nation's women's knowledge (Chapola et al. 2024; Datta et al. 2024) demonstrate how gender inclusive adaptation and resilience might be enacted. The latter is particularly important. For Indigenous Peoples and culturally diverse communities—who were not a specified focus population in the present review—the degradation of ecosystems and biodiversity not only disrupts physical environments but can also erode cultural continuity, spiritual wellbeing and collective identity, amplifying the gendered and racialised impacts of environmental harm (van Daalen et al. 2024). For example, in rural Fiji, the loss of important totem species, due in part to a succession of cyclone events, has far‐reaching intergenerational impacts as cultural traditions that involve gendered rituals are lost, and related collective identities are impacted (Singh‐Peterson 2025).

A recent study on the climate justice experiences of nurses and community partners found that a colonial view of humanity's separation from nature leads to desensitisation to gendered violence against Indigenous Peoples and all life on Earth (LeClair et al. 2025). Participants noted that colonialism and cis‐heteropatriarchy are interconnected systems of domination, particularly in Indigenous communities that respect gender‐diverse Two‐Spirit individuals and struggle against Eurocentric gender and sexual binaries, cultural erasure, genocidal policies and land loss. In the context of the Triple Planetary Crisis, recognising and responding to both social and cultural determinants is essential to advancing equity, environmental justice, and gender justice in an interconnected and sustainable way.

### 5.7 Nursing Role

In response to the Triple Planetary Crisis, nurses are uniquely positioned to advance gender justice for two interrelated reasons. Firstly, nurses are the largest and most geographically dispersed health workforce globally. Nursing's long‐standing tradition of advocacy positions the profession not only as a witness to environmental injustice but as credible and experienced advocates for gender‐responsive systemic change. Secondly, nursing is a profession whose workforce predominantly identifies as women, historically shaped by structural gendered inequities including the undervaluation of care work, barriers to leadership, constrained professional autonomy, and limited influence in policy decision‐making (Salvage and White 2020). These dynamics mirror the gendered power imbalances exacerbated by the Triple Planetary Crisis presented in this review (Figure 7).

Operationalising this role requires nurses to move beyond a narrow biomedical framing of environmental impacts and engage with the gendered social determinants of vulnerability and health. In practice, nurses can integrate gender‐sensitive environmental risk assessments into routine care, accounting for factors such as unpaid care burdens, occupational exposure, food and water insecurity, geographical displacement, and heightened risks during pregnancy and disaster contexts. In education, nursing curricula at all levels should embed environmental and gender justice as core competencies (Levett‐Jones et al. 2023; Nicholas and Breakey 2017). At the policy level, nursing professional bodies and unions can leverage their collective authority to advocate for gender justice in policy, including recognition of care work, workforce protections and equitable representation in decision‐making forums. Guided by nursing's history and underpinned by the International Council of Nurses Code of Ethics (International Council of Nurses 2021) and recently updated definition of nursing and nursing work (White et al. 2025), these strategies position nurses as central, and effective actors in advancing gender justice amidst Triple Planetary Crisis.

### 5.8 Gaps in the Literature

Two notable gaps were identified in the literature, which constrained the extent to which all research questions could be fully addressed. First, none of the included studies reported on the experiences of gender diverse populations, including transgender and non‐binary individuals in the context of the Triple Planetary Crisis. This should not be taken as evidence that such impacts do not exist. On the contrary, emerging reports highlight that LGBTQIA+ communities often face heightened risks due to poverty, housing insecurity and discrimination in disaster response (Mann et al. 2024). Thus, the lack of academic discourse might reflect a gap in research funding and processes, rather than the absence of a problem.

This scoping review also identified a significant gap in the literature exploring the gendered experiences of health professionals in the context of the Triple Planetary Crisis. The experience of nurses, in particular, has been underexplored in contemporary literature. This is surprising, as nurses may indeed have considerable insight into how women are experiencing the Triple Planetary Crisis because they themselves are a part of a gendered profession (World Health Organization 2025). While efforts to achieve greater gender balance in the nursing profession are essential, it remains crucial to acknowledge that nursing is inherently shaped by gendered dynamics. Approximately 87% of the global nursing workforce identify as women (World Health Organization 2025). Further, the Triple Impact of Nursing report suggests that the way nurses are treated within health settings often reflects how women are treated in society (All‐Party Parliamentary Group on Global Health 2016). The report highlights how broader gender norms, particularly across cultures where health labour work is often undervalued, seen as low‐status work, or poorly paid (Langer et al. 2015), mirror how women's economic and social contributions are valued (All‐Party Parliamentary Group on Global Health 2016; Lingel et al. 2022).

Existing policy and societal responsiveness to environmental threats may further reveal how little women's lives and labour are valued (Crenshaw 1991; Gaard 2015). For example, although health workers are expected to lead the frontline response and are often victims of extreme weather events themselves, less than 5% of climate change adaptation funding is directed to health, with under 1% of multilateral climate finance specifically supporting health‐related projects (World Health Organization 2018).

While women make up the bulk of the health workforce, they are also consistently underrepresented in executive or management roles across the health system (Langer et al. 2015). Nurses in particular are underrepresented in climate adaptation and resilience policymaking, detrimentally impacting their adaptive capacity (Richards 2025). This sentiment parallels a key finding of this Scoping Review, which was that women were reportedly unrepresented or subdued in domestic (Jannat and Kusakabe 2024; Obossou et al. 2023; Wood et al. 2021), local community (Andajani‐Sutjahjo et al. 2015; Goli et al. 2023; Obossou et al. 2023), national (Hlahla et al. 2022) and international decision‐making (Hlahla et al. 2022). The exclusion of women and nurses from leadership and strategic planning not only compromises workforce protection and preparedness, it positions women healthcare workers at the frontline of risk without adequate voice, agency, or structural support (Langer et al. 2015). As women (including many health workers) face increasing impacts from environmental degradation, the lack of focused attention may suggest a gender bias that warrants further exploration.

Strengthening women's property rights (Segnestam 2017; Sommer et al. 2022; Wood et al. 2021) is an important goal for advancing gender equity. Investments in women's access to financial resources and strengthened property rights were associated with both greater empowerment (Ghasemi et al. 2021) and enhanced environmental conservation (Ghasemi et al. 2021) – illustrating how supporting women can simultaneously drive social and environmental gains (Sommer et al. 2022).

## 6 Recommendations

Advancing gender justice responses requires first acknowledging that women, gender‐diverse people and those working in female dominated healthcare roles remain marginalised within discourses on gender equity in the context of the Triple Planetary Crisis (Langer et al. 2015; van Daalen et al. 2024). There is a need for research that illuminates and examines these experiences more fully, in order to identify solutions that promote gender justice.

Secondly, pathways toward gender equity must continue to be promoted. This is not a separate or new agenda, but rather a refreshed commitment under the auspices of effective environmental policy. The findings of this scoping review should not be taken as a blanket prescription; they must be interpreted within the context in which they are set. Countries should therefore pursue independent research into culturally specific impacts and pathways to inform more nuanced gender justice responses.

Finally, healthcare workers, including nurses, can support gender‐responsive environmental action by staying abreast of the health challenges experienced by women and gender‐diverse people, seeking to improve women's access to care (Dutta et al. 2024), and advocating for inclusive environmental policies (Ghosh and Dutta 2024; Patrick and Capetola 2011). Strengthening gender‐sensitive communication, data collection and service delivery may contribute meaningfully to more equitable health systems. Additionally, strengthening public health systems by improving infrastructure (Austin et al. 2021), increasing staffing (Austin et al. 2021) and considering gender‐responsive planning (Hlahla et al. 2022; Puwajarernpong et al. 2023) are critical to protecting women and gender‐diverse people from the health impacts of the Triple Planetary Crisis.

## 7 Limitations

Climate change dominated the evidence base relative to pollution and biodiversity loss, which may constrain the breadth of cross‐domain interpretation.

Studies reporting incidental health findings based on sex or gender were excluded unless they explicitly engaged with gender justice, as defined by our guiding framework. This may have narrowed the scope of included evidence and limited capture of implicit or emergent gendered inequities; however, inclusion of such granular evidence from a vast and disparate body of literature would have been beyond the limited time and resources of our project. Further, due to the nonspecific and often amorphous or undetailed mention of such evidence, cataloguing and deriving cogent conclusions would have been extremely challenging and unlikely to yield clear results or potentially impossible. Additionally, much of the existing literature focuses on individuals assigned female at birth, reflecting primarily the experiences of cisgender women. Consequently, the intersectional experiences of transgender and non‐binary individuals are thus underrepresented. This constrains the applicability of findings across diverse gender identities.

While not constrained by geographic limitations, this review did not seek to specifically include or report on Indigenous Peoples and people from culturally and linguistically diverse groups, or those from migrant backgrounds. These diverse groups, as noted above, are often unequally impacted by the Triple Planetary Crisis, and individual capacities to cope, adapt and transition away from risks vary greatly from setting to setting.

Another limitation is that while temporality was originally included as a component of our gender justice framework, it was removed during the review process due to a lack of findings related to this concept. This restricts understanding of how gendered impacts and pathways may evolve over time. Furthermore, there were several studies that could not be retrieved during the search. While targeted retrieval strategies such as author contact or interlibrary loan may increase completeness, such approaches were not undertaken due to time constraints. Given the small number of unretrieved records relative to the total screened, the impact on the overall mapping of evidence was deemed limited by the research team. In addition, this review did not include grey literature and was limited to English‐language publications, consistent with its focus on mapping peer‐reviewed empirical evidence; however, the inclusion of grey literature may be valuable for future analyses seeking more localised, practice‐based, or policy‐specific insights.

Finally, the geographical origins of the included studies are predominantly from low‐income countries which may limit generalisability. This may bias interpretations toward contexts with distinct socio‐economic and governance conditions. Thus, the findings must be considered in the context of these constraints and further work should be conducted in contexts where evidence is currently limited.

## 8 Conclusion

This scoping review elucidated the intersecting dynamics that continue to reinforce systemic gender inequities, restricting women's full participation across social, economic and political spheres. These entrenched inequalities not only heighten vulnerability to environmental crises but also constrain their capacity to adapt. The perspectives of nurses and gender diverse people, whilst currently missing in the literature, are critical to understanding the gendered impacts of the Triple Planetary Crisis and moving toward gender justice. Until the voices of women, gender‐diverse people and nurses are meaningfully recognised and integrated, achieving gender justice within healthcare will remain an aspirational rather than attainable goal.

## Funding

This research was supported by the Commonwealth through an Australian Government Research Training Program Scholarship.

## Ethics Statement

As this study is a literature review, no primary data were generated or analysed, and all sources used are publicly available and properly cited within the manuscript. Given the nature of the research, ethics approval and patient consent were not required, as no human participants were involved. Additionally, no permissions were needed for reproduced material. This research was supported by the Australian Government Research Training Program (RTP) Scholarship. I declare no conflicts of interest related to this study.

## Conflicts of Interest

The authors declare no conflicts of interest.

## Data Availability

Data sharing not applicable to this article as no datasets were generated or analysed during the current study.

## Appendix A Ovid Medline Search Strategy

Ovid MEDLINE(R) ALL.

1 Planetary health. mp. 1778

2 Climate change.mp. 89,635

3 Climate action. mp. 858

4 Climate resiliency. mp. 34

5 Climate justice. mp. 234

6 environmental health.mp. 26,770

7 Anthropocene.mp. 1743

8 Anthropogenic.mp. 46,078

9 environmental responsibility.mp. 273

10 nature‐relatedness.mp. 111

11 Pro‐environmental behaviour. 128

12 climate activism.mp. 34

13 carbon emission.mp. 3753

14 environmental sustainability.mp. 6804

15 global warming.mp. 19,719

16 (sustainab* adj3 environment*).mp. 15,822

17 ‘environmental responsibility’.mp. [mp = title, book title, abstract, original title, name of substance word, subject heading word, floating sub‐heading word, keyword heading word, organism supplementary concept word, protocol supplementary concept word, rare disease supplementary concept word, unique identifier, synonyms, population supplementary concept word, anatomy supplementary concept word] 273

18 Biodiversity loss.mp. [mp = title, book title, abstract, original title, name of substance word, subject heading word, floating sub‐heading word, keyword heading word, organism supplementary concept word, protocol supplementary concept word, rare disease supplementary concept word, unique identifier, synonyms, population supplementary concept word, anatomy supplementary concept word] 2842

19 ‘Environmental degradation’.mp. [mp = title, book title, abstract, original title, name of substance word, subject heading word, floating sub‐heading word, keyword heading word, organism supplementary concept word, protocol supplementary concept word, rare disease supplementary concept word, unique identifier, synonyms, population supplementary concept word, anatomy supplementary concept word] 4110

20 ‘Pollution’.mp. [mp = title, book title, abstract, original title, name of substance word, subject heading word, floating sub‐heading word, keyword heading word, organism supplementary concept word, protocol supplementary concept word, rare disease supplementary concept word, unique identifier, synonyms, population supplementary concept word, anatomy supplementary concept word] 231,841

21 ‘Climate crisis’.mp. [mp = title, book title, abstract, original title, name of substance word, subject heading word, floating sub‐heading word, keyword heading word, organism supplementary concept word, protocol supplementary concept word, rare disease supplementary concept word, unique identifier, synonyms, population supplementary concept word, anatomy supplementary concept word] 1100

22 ‘Ecological crisis’.mp. [mp = title, book title, abstract, original title, name of substance word, subject heading word, floating sub‐heading word, keyword heading word, organism supplementary concept word, protocol supplementary concept word, rare disease supplementary concept word, unique identifier, synonyms, population supplementary concept word, anatomy supplementary concept word] 164

23 ‘Global warming’.mp. [mp = title, book title, abstract, original title, name of substance word, subject heading word, floating sub‐heading word, keyword heading word, organism supplementary concept word, protocol supplementary concept word, rare disease supplementary concept word, unique identifier, synonyms, population supplementary concept word, anatomy supplementary concept word] 19,719

24 Environmental Justice.mp. [mp = title, book title, abstract, original title, name of substance word, subject heading word, floating sub‐heading word, keyword heading word, organism supplementary concept word, protocol supplementary concept word, rare disease supplementary concept word, unique identifier, synonyms, population supplementary concept word, anatomy supplementary concept word] 1873

25 “Triple planetary crisis”.mp. [mp = title, book title, abstract, original title, name of substance word, subject heading word, floating sub‐heading word, keyword heading word, organism supplementary concept word, protocol supplementary concept word, rare disease supplementary concept word, unique identifier, synonyms, population supplementary concept word, anatomy supplementary concept word] 15

26 1 or 2 or 3 or 4 or 5 or 6 or 7 or 8 or 9 or 10 or 11 or 12 or 13 or 14 or 15 or 16 or 17 or 18 or 19 or 20 or 21 or 22 or 23 or 24 or 25 396,581

27 anthropogenic effects/or carbon sequestration/or climatic processes/or exp. climate change/or global warming/or sea level rise/or cyclonic storms/or droughts/or floods/or greenhouse effect/or tidal waves/or carbon footprint/69507

28 gender equity/or intersectional framework/or feminism/or reproductive rights/or right to health/or social justice/or environmental justice/or women's rights/28276

29 gender identity or femininity or transsexualism 27335

30 exp. “sexual and gender minorities”/or vulnerable populations/35980

31 health personnel/or community health workers/or emergency medical technicians/or home health aides/or licensed practical nurses/or exp. nursing assistants/or operating room technicians/or paramedics/or physical therapist assistants/or anaesthetists/or audiologists/or case managers/or faculty, medical/or faculty, nursing/or health educators/or exp. health facility administrators/or exp. medical staff/or exp. nurses/or exp. nursing staff/or nutritionists/or occupational therapists/or optometrists/or pharmacists/or physical therapists/or physician executives/or exp. physicians/or psychotherapists/527860

32 Health professionals.mp. [mp = title, book title, abstract, original title, name of substance word, subject heading word, floating sub‐heading word, keyword heading word, organism supplementary concept word, protocol supplementary concept word, rare disease supplementary concept word, unique identifier, synonyms, population supplementary concept word, anatomy supplementary concept word] 70,563

33 ‘Healthcare workers’.mp. [mp = title, book title, abstract, original title, name of substance word, subject heading word, floating sub‐heading word, keyword heading word, organism supplementary concept word, protocol supplementary concept word, rare disease supplementary concept word, unique identifier, synonyms, population supplementary concept word, anatomy supplementary concept word] 27,170

34 ‘Nurse’.mp. [mp = title, book title, abstract, original title, name of substance word, subject heading word, floating sub‐heading word, keyword heading word, organism supplementary concept word, protocol supplementary concept word, rare disease supplementary concept word, unique identifier, synonyms, population supplementary concept word, anatomy supplementary concept word] 211,061

35 ‘Nursing’.mp. [mp = title, book title, abstract, original title, name of substance word, subject heading word, floating sub‐heading word, keyword heading word, organism supplementary concept word, protocol supplementary concept word, rare disease supplementary concept word, unique identifier, synonyms, population supplementary concept word, anatomy supplementary concept word] 690,181

36 ‘Doctors’.mp. [mp = title, book title, abstract, original title, name of substance word, subject heading word, floating sub‐heading word, keyword heading word, organism supplementary concept word, protocol supplementary concept word, rare disease supplementary concept word, unique identifier, synonyms, population supplementary concept word, anatomy supplementary concept word] 107,781

37 ‘Medical staff’.mp. [mp = title, book title, abstract, original title, name of substance word, subject heading word, floating sub‐heading word, keyword heading word, organism supplementary concept word, protocol supplementary concept word, rare disease supplementary concept word, unique identifier, synonyms, population supplementary concept word, anatomy supplementary concept word] 43,528

38 ‘Midwives’.mp. [mp = title, book title, abstract, original title, name of substance word, subject heading word, floating sub‐heading word, keyword heading word, organism supplementary concept word, protocol supplementary concept word, rare disease supplementary concept word, unique identifier, synonyms, population supplementary concept word, anatomy supplementary concept word] 24,262

39 ‘public health’.mp. [mp = title, book title, abstract, original title, name of substance word, subject heading word, floating sub‐heading word, keyword heading word, organism supplementary concept word, protocol supplementary concept word, rare disease supplementary concept word, unique identifier, synonyms, population supplementary concept word, anatomy supplementary concept word] 510,657

40 ‘allied health’.mp. [mp = title, book title, abstract, original title, name of substance word, subject heading word, floating sub‐heading word, keyword heading word, organism supplementary concept word, protocol supplementary concept word, rare disease supplementary concept word, unique identifier, synonyms, population supplementary concept word, anatomy supplementary concept word] 27,046

41 32 or 33 or 34 or 35 or 36 or 37 or 38 or 39 or 40 1,468,982

42 31 or 41 1,741,434

43 26 or 27 419,801

44 28 or 29 or 30 84,896

45 42 and 43 and 44 375

## Appendix B Systemic Oppression and Power Dynamics

**Table:**

Subtheme	Subcategory(ies)	Description	Key quotes and examples
Abuse *n* = 1	Pacification Sexual Assault Violence	Characterised in the literature by women's experiences of gendered violence at protests, where intimidation and sexual assault was used by police officials as a means of pacification and subjugation.	‘I did have an officer so close behind me, his entire body was pressed against mine the entire time … And because I was walking the slowest, he was pressed right into me and just walked me the entire road … I did tell him all the time, I would try [to move] to one side or another side, he just stayed exactly almost glued to my back, and it felt very, very violating, very violating’ (Monk et al. 2019) ‘He purposely touched my left breast and squeezed it. You know when you've been touched inappropriately, don't you? You have a reaction to it. So I said, “You've just touched me. You've just touched me sexually. You've just sexually assaulted me”. And he just smiled at me and his colleague was with him. I said, “You saw him do that”. And his colleague was smiling at me as well.’ (Monk et al. 2019) ‘Some women at BMCPC felt violated, frightened and fearful, and this violence had a direct effect on how camp residents and supporters engaged with the protest.’ (Monk et al. 2019)
Education *n* = 8	Access to education	Access to education for many women continues to be a challenge.	‘Rural women's and men's ability to perceive climate change depends on their access to relevant information from government extension services as well as farmers' groups’ (Floro et al. 2019)
		Women with suboptimal access to education also then face diminished opportunities for meaningful employment free of exploitation.	‘Results indicated that percentage of women (25 and older) with some secondary education had an inverse relationship with the estimated prevalence of modern slavery across countries’ (Cameron et al. 2021)
	Climate literacy	While some women had good climate literacy, those who do not may experience suboptimal climate change adaptive capacity.	‘I believe women are more concerned and knowledgeable about environmental change. For example, traditionally, I have seen chiefly women planting and worshipping Tulsi (Basil), Peepal ( *Ficus religiosa* ), and Vat (Banyan) trees in my community. (a 34‐year‐old male) Here in my village, it is usually females who usually collect wood and grass for domestic animals and other daily chores. Therefore, they have more excellent environmental knowledge and are more concerned about climatic change. (a 48‐year‐old female)’ (Das 2024) ‘The knowledge of climate change is low among the respondents, but they accepted the reality of it when the phenomenon was explained to them. In the words of a respondent; Climate change? I have not really heard about that. But with your explanation, I can say that climate change has been affecting us in Ilaje because the waters that floods our streets, houses and shops have been increasing year by year.’ (Ojakorotu and Olajide 2018) The women, themselves, also have low adaptive capacity. The primary reasons for this include a lack of knowledge and resources and a sense of helplessness to deal with the impacts of climate change and variability. (Hlahla et al. 2022).
Financial oppression (*n* = 19)	Access to credit and/or finances	There were examples of women, particularly young women, not having equitable access to finances (including personal finances and loaning money).	‘More male‐headed households belong to a cooperative organisation and have access to credit than female‐headed households do.’ (Obossou et al. 2023) ‘Since men were most commonly identified as the owners of the land and the houses that, unless one had access to a guarantor, were required as collateral, men had much better capacity to borrow money.’ ‘The study further revealed that men's control over financial resources in the household is based on a social order that gives household heads (usually males) power over others even in circumstances where the women may be contributing considerably to the survival of the home. These gender power relations in regards to control over financial resources mean that women have to seek curative or preventive health care services under the direct permission of men.’ (Shuaib et al. 2019) ‘Elder women are able to control their income and use their earnings to offset food shortages after food stores are depleted. Young women are not granted the power to use their earnings to respond to their households' food insecurity’ (Wood et al. 2021)
	Access to material resources	Women sometimes lacked access to ownership or decision making for material resources such as land and water, which made income generation difficult. This was a particularly pertinent theme across cultural contexts where subsistence agriculture was common.	‘Their susceptibility is increased by their restricted access to resources including financing, extension services, land ownership, and agricultural inputs’ (Das 2024) ‘Women's activities are closely related to water, and women therefore are the first ones who often note changes in water quality or other water related problems’ (Andajani‐Sutjahjo et al. 2015) ‘Men may have better access to resources like livestock, which produce manure, or more money to buy fertilisers’ (Obossou et al. 2023) ‘The female heads of household had access to fewer and smaller animals, such as chickens and pigs, while the women and men in the male‐headed households often mentioned larger animals as well, such as cows, heifers, or oxen. Ownership of the larger animals was, however, commonly assigned to the men’(Segnestam 2017)
	Socio‐economic disparity and climate change vulnerability	Disparity between women and men's access to resources and finances impacted on climate change vulnerability.	‘Results indicate that gender disparities in CC vulnerability not only reflect preexisting gender inequalities but also reinforce them. Inequalities in ownership and controlling household assets and rising familial burdens due to male out‐migration, declining food and water access, and increased disaster exposure can undermine women's ability to achieve economic independence, enhance human capital, and maintain health and well‐being’ (Vésier and Urban 2023)
Governance, leadership, policy and decision making (*n* = 15)	Access to community groups and forums	Participation in community forums and groups creates a network of actors that can work collectively to respond to environmental threats. Women did not always have access to such community forums.	‘More male‐headed households belong to a cooperative organisation and have access to credit than female‐headed households do.’ (Obossou et al. 2023) ‘Women's social capital is more vulnerable than men's during climate crisis due to higher participation in social networks.’ (Goli et al. 2023)
	Political exclusion	Women are not always proportionately represented in various political forums.	‘Thai hierarchies of social participation have arguably impeded women's chances to participate in politics. Individual and group discussion with women village administrators and leaders confirmed the dominant perception of female leaders being inferior to male leaders.’ And ‘…a female leader has to work twice as hard as her male counterpart to prove herself so that she can do her job as well as the male leader. In order to achieve the same quality or amount of work, a man does 5 things and a woman needs to do 10 things. Thus we see more women become assistants of male bureaucrats than leaders.’ (Andajani‐Sutjahjo et al. 2015) ‘Women are inexplicably underrepresented in national and international climate change discourses and negotiations and in the implementation of measures for facilitating mitigation and adaptation’ (Hlahla et al. 2022) ‘Many co‐researchers have argued that Indigenous people (particularly women) are not included in emergency management and other efforts to adapt to changes’ (Chapola et al. 2024)
	Decision making	Women are often excluded from making decisions and processes that shape climate responsive policies.	‘Within households, women are often excluded from decision‐making processes that help mitigate climate‐stressed food production’ (Wood et al. 2021) ‘I complained but no one was listening. I want an improved water quality… but I don't know what to do and how’ (Andajani‐Sutjahjo et al. 2015) ‘The study findings show that in most households, women play a significant role in food choices. However, the male member of the household dominates in the decision‐making due to their greater social position in the traditional community.’ (Das 2024) ‘In the process of CBA [community‐based adaptation’] activities, women do not influence the organisation's decisions, nor do they facilitate local discussions to identify the needs of their women members. Women have limited options to influence CBA decisions.’ (Jannat and Kusakabe 2024) ‘Structural barriers, including lack of access to and control over productive resources and agricultural services, time constraints resulting from unpaid domestic chores, inequality in decision‐making, and restricted mobility, all frequently account for gender gaps in crop productivity’ (Obossou et al. 2023)
Labor (*n* = 13)	Workforce participation	Women participate in the workforce. Their contribution is not always recognised, sometimes undervalues and in some cases exploited under modern slavery arrangements.	‘Women's human capital was more at risk than that of men. This is because the importance of women's role, despite their activities in agriculture, food security, caring responsibilities, housekeeping and motherhood, has been underestimated for a long time.’ (Goli et al. 2023) ‘Results indicated that percentage of women (25 and older) with some secondary education had an inverse relationship with the estimated prevalence of modern slavery across countries (*β* = −0.146).’ (Cameron et al. 2021)
	Domestic duties	Many articles identified that women were expected to perform domestic duties, often unpaid, and often to the detriment of earning income from work performed elsewhere.	‘The important question is, how much time and effort does it take for a woman to fulfil each role? In the case of the female leaders of the Prik Tai community, they use their free time from earning money and taking care of the family and the household to work in the community.’ (Puwajarernpong et al. 2023) ‘Young women are expected to perform most of the daily household tasks, often at the direction of elder women. This suggests that elder women and young women are navigating different climate‐linked realities, despite occupying the same spaces.’ (Wood et al. 2021) ‘Women are also the most exposed to climatic shocks because of the great responsibilities they often have with families.’ (Asongu et al. 2022) ‘Patriarchal systems are reinforced by the adoption of the notion that cooking is only the domain of women. Because they devote a disproportionate amount of their time and energy to household responsibilities, women's prospects for personal and professional growth outside the home are limited. “*Due to the old customary norms, I have to cook and serve meals to the whole family. Even my mother has told me the same; it is a women's duty to cook in the kitchen”—*(46‐year‐old female)’ (Das 2024) ‘Most women take care of the household and do farming, but they are considered to be their husbands' helpers.’ and ‘Their problem is that they are responsible for both productive and reproductive work and are too busy to participate. The social expectations for Rakhine women are such that women are supposed to do all the work, so they get little support from their men.’ (Jannat and Kusakabe 2024)
	Reproductive and caring work		‘Reproductive work in all households was mostly the responsibility of females. The only exception to this was in the house of an amputee, who was unable to work. In this instance, men had to take care of the house’ and ‘In summary, women are involved in both roles in the house, production and reproduction. By contrast, men play their largest role in productive jobs’ and ‘At the household level before disasters, women are responsible for collecting belongings, preparing food and drinking water, and caring for family members. During a disaster, whether in the households of community leaders, community members, or vulnerable groups, women still manage the house.’ (Puwajarernpong et al. 2023) ‘participants [women] recognised that responsibility for keeping them [offspring] “safe” from smoke exposure fell primarily to them’ (Allen et al. 2022)
Mobility	Restricted mobility	Inability to exercise freedom in movement throughout the community. The literature reports that culture and child rearing responsibilities were factors influencing their mobility.	‘In the cases of Muslim women, their sense of insecurity comes from restricted mobility due to religious conservatism. Muslim women need to observe purdah, their family members do not allow them to attend meetings or talk to men (NGO facilitators are often men), and if they do, they need to cover their faces and keep their profile low.’ and ‘For living, they are obliged to go outside the house. However, their mobility is limited to the Rakhine market and within the community since they fear teasing because of their dress patterns and language.’ (Jannat and Kusakabe 2024) ‘The women's group observed that they mainly use walking and omni‐buses to balance child rearing and commercial responsibilities, although a few own private cars, especially among higher income groups.’ (Shuaib et al. 2019)
Health	Susceptibility to illness	Gendered differences in susceptibility to certain illnesses may arise due to intersecting biological and sociocultural determinants.	‘Women and children are more susceptible to hunger, waterborne illnesses, and other health problems associated with environmental degradation and low water quality as a result of climate change.’ and ‘In traditional families, women still follow the customary norms of not eating before male members. A 56‐year old female respondent said that women still eat at the end, i.e., when male family members finish eating, due to old customary values. Sometimes, they do not get enough, affecting women's nutrition and food security. Therefore, at the time of extreme climatic conditions, the scarcity of resources further hamper the nutritional security of women due to still prevalent gender‐defined norms of eating habits.’ (Shuaib et al. 2019)

## Appendix C Differing and Layered Impacts

**Table:**

Subtheme	Subcategory(ies)	Description	Key quotes and examples
Health (*n* = 16)	Health impacts	Women experienced health impacts arising of environmental threats including impacts of: mental illness, anxiety, stress, contaminated water supplies, food insecurity and malnutrition, fatigue, gynaecological disease, heat stress, physiological injury, sexually transmitted diseases, sleeplessness and mortality.	‘On average, the proportion of women reporting occurrence of illness was significantly higher than men at 36 and 26% respectively.’ (Amondo et al. 2022) ‘Young mothers from both upper castes and tribal communities are getting increasingly depressive, anxious and tense with recurrent disasters, loss of property, destruction of homestead and the challenges of rebuilding lives and livelihoods along with burden of other diseases. (Ghosh and Dutta 2024).’ ‘Many Indigenous women (co‐researchers) highlighted the additional layer of mental stress, mood swings, emotional disorders, etc., experienced by Indigenous women and community members who are already facing significant changes due to the loss of land‐based activities, their ancestral territories and their knowledge systems’ (Chapola et al. 2024) ‘Respondents commented that the increasing temperatures make them lethargic, decreasing their work output and 23% complained that climate change and variability is affecting their general well‐being and causing them discomfort’ (Hlahla et al. 2022) ‘Water is becoming a scarce resource within these communities, causing a few respondents (7%) to be fearful that they may have to purchase water in the future, further diminishing their incomes.’ (Hlahla et al. 2022) ‘Traditionally, the daily meals comprised fish and rice in the region, with occasional pulses (lentils). Respondents revealed that edible fish were no longer available in the brackish waters, canals, shallow rivers rivulets that compose the “commons” in the Sundarbans. Lentils also do not grow anymore because the soil is too saline. Even rice, a staple food in the region, has gradually become harder to cultivate because of rising salinity, claimed local farmers.’ (Ghosh and Dutta 2024) Disaster management focuses on saving human lives but death of livestock snowballs into long‐term negative impacts on household health, particularly that of the children,” said a grandfather in Ghoramara Island in western Sundarbans. (Ghosh and Dutta 2024) Our results also suggest that food insecurity has indirect impacts through women's socio‐health status. Specifically, we find food insecurity negatively impacts women's socio‐health status, including education, fertility, and access to medical care (−0.53)0.5 Women's socio‐health status in turn influences use of contraceptives (0.33), which is directly associated with lowering the percent of the adult population living with HIV who are women (−0.52) in less‐developed countries. (Austin et al. 2021) ‘Women were found generally more at risk to die because of heat than men, and the difference was larger among people over 85 years old. Risks among married people were lower than risks among single, divorced, and widowed people, while risks in divorced women were significantly higher than in divorced men’ (Vésier and Urban 2023) ‘In terms of human capital (assets), the most important subcategory for men (*n* = 23) was food security (f = 12). However, for female‐headed households (*n* = 23), the most important human capital (assets) issues were food security, mental health, mortality and illness, and adverse effects on children's and older people's health (f = 13). They believed that with the onset of these crises, access to safe and accessible food would be restricted, and, as a result, physical injuries and diseases caused by a lack of adequate and healthy food would occur’ (Goli et al. 2023)
	Health system and services	Access to health services was impacted by weather events.	‘Ontime access to health centers is additionally constrained by flooded community access roads.’ (Shuaib et al. 2019)
Financial Impacts *n* = 13	Impact on economic rights	Changes in extreme weather event patterns had implications for women's access and expression of economic rights.	‘When the disaster variable moves from 0 to 3 (major disaster deaths), we observe that the predicted probability of major respect for women's economic rights declines by 93% (from 0.003 to 0.0002). The size of the decline in the probability of respect for women's economic rights is an indication that the hypothesized effect of disaster events is substantively significant in affecting women's economic status.’ (Detraz and Peksen 2017)
	Impacts on capital	One study identified that the impact on capital differed between men and women, with men experiencing heightened vulnerability to their financial capital, and women experienced greater risk to human capital.	‘Since the income from the agricultural activities of the majority of female‐headed households was significantly different from that of male‐headed households, and men had better financing than women, the comparisons of the mean responses of men and women showed that the men's financial capital (X^−^ = 12.5) was more vulnerable to climate crises than that of women (X^−^ = 10.75)’ (Goli et al. 2023) And ‘Women's human capital was more at risk than that of men. This is because the importance of women's role, despite their activities in agriculture, food security, caring responsibilities, housekeeping, and motherhood, has been underestimated for a long time.’ (Goli et al. 2023)
	Impact on work	Climate related extreme weather events impacted on women's ability to work and generate income. This was not always a gendered finding, as both men and women experienced differing impacts on their work. In some instances, women needed to work harder and in unsafe conditions due to extreme weather events.	‘Respondents are of the opinion that rainfall is more intensive which brings with economic challenges to women due to lack access to places of economic activities’ (Ojakorotu and Olajide 2018) And ‘In terms of how climate change constitutes economic challenge to women in coastal communities, the extended rainfall ensures that women are denied access to places of work such as their stalls and shops. This directly leads to usually long period in which women are not able to carry on economic activities through which they feed their families and unable to meet up with financial responsibilities which include payment of house rents, school fees of children, purchasing of food stuff and among others.’ (Ojakorotu and Olajide 2018) ‘During the summer season, the water source is dried, so the alternative village is the only option, which is far away from my house. Even sometimes, we have to manage daily chores with a single bucket of water’ (a 34‐year‐old female) (Ojakorotu and Olajide 2018) ‘The severe and adverse effects of CC [climate change] not only affect women's livelihood opportunities but also increase their workload and fatigue while lowering their self‐ esteem, forcing them to engage in dangerous activities.’ (Goli et al. 2023)
	Impact on income and poverty	In some instances, environmental damages and subsequent loss of income rendered women vulnerable to poverty.	‘According to young mothers, multiplying sources of income has become an absolutely essential responsibility for them as agriculture and fishing fail to yield, disaster losses accrue, and newer expenses (out‐migration) arise’ (Ghosh and Dutta 2024) ‘One of the respondents believed that: ‘…climatic disasters have a direct impact on people's social activities because of reducing their income’ (Goli et al. 2023) ‘My house was broken due to flood; I couldn't afford to put a plumbing system and fix the house. I was struggling to put food on my table and managing other expenses. Asa young single mother with four kids I went through unfair, discriminatory, helpless, and stressful situations and lost hope many times’ (Chapola et al. 2024)
Safety and Security *n* = 10	Risky coping mechanisms	To withstand some of the impacts of climate change on work and financial security, women engaged in more risky, unsafe work and health practices.	‘A decrease in household income can result in the participation of women in risky behaviours such as transactional sex for food or income’ (Hlahla et al. 2022) ‘Those who cannot join crab‐catching trips, resort to collecting prawn seedlings from the rivers. “It is very strenuous activity. For over six hours I walk in chest‐deep water on the riverbed and swim consistently, holding the fishing‐net tight, to get a decent catch. I feel completely exhausted in the afternoon but it's some additional income to buy a health drink or biscuits for my children,” said a 40‐year‐old woman in Sandeshkhali II village. But IHPs point out another grave risk that women fail themselves to recognise. Apart from the physical exhaustion, this practice makes women vulnerable to cervical cancer and other gynaecological diseases.’ (Ghosh and Dutta 2024) ‘Workers ascribed the working conditions to institutionalised racism in the south generally, and to the agricultural industry specifically. Many respondents used terms such as “slave‐like”’ (Saville and Adams 2021) ‘When asked, the person said that the women needed the support in calming their minds and to sleep well. “Hardships have increased manifold, it is simply too much for them to handle. They need the help of these medicines,” he said. However, he was neither licensed to prescribe such medications nor should he have access to them.’ (Ghosh and Dutta 2024)
	Increased exposure to violence		‘The policing at Barton Moss was experienced as a systematic abuse of power and an attempt to curtail the rights of women to protest. Here, police power plays on the productivity and effectivity of sexual violence—long tested as the most efficient way to control women and to unsettle their occupation of public space’ (Monk et al. 2019) ‘The odds of respondents reporting any form of intimate partner violence were more likely among women who lived in regions that experienced severe weather events than those who did not live in regions that experienced severe weather events’ (Munala et al. 2023)
Impact on women as mothers *n* = 8	Care giver burden		‘These feelings of anxiety were accompanied by public health advice which firmly placed responsibility for reducing (un)born children's smoke exposure on women, by suggesting those who were pregnant wear masks and remain indoors. As a consequence, mothers in our study vigilantly checked air quality apps to measure smoke toxicity and adapted daily activities accordingly.’ and ‘Feeling “completely responsible” as Sarah puts it, for the air their children breathe, is produced through a set of bushfire intra‐relations that saddle women with responsibility’ (Allen et al. 2022)
	Concern for children	Food insecurity and air quality	‘Inability to offer “good food” to their children produces a strong sense of guilt and emotional stress in mothers. “I feel terrible offering only rice and onions, sometimes potatoes, to my children and parents. My eldest son is a teenager, he feels always hungry.’ (Ghosh and Dutta 2024) ‘It is unsurprising that participants expressed considerable concern for their unborn babies as seen in the following narratives. … It was really constantly on my mind, and I tried to kind of not get too anxious about it, but it was really difficult because… I mean, you just think about it all the time. You're just constantly worrying when you're pregnant what's going to affect the baby. Like everything you do. (Alice, pregnant during fires)’ (Allen et al. 2022)
Impact on spiritual wellbeing and practice *n* = 4	Indigenous and traditional practices	Articles described the impact of environmental threats on cultural practices.	‘The intricate web of climate change extends to family relations, community cohesion, Indigenous culture and intergenerational learning’ (Chapola et al. 2024) ‘Climate crisis impacts extend beyond environmental concerns for Indigenous women, encompassing threats to livelihoods, land‐based cultures, and the intricate relationships between people and nature.’ and ‘Colonisation has destroyed the natural pre‐laws and created historical injustice and vulnerability/exposure of the indigenous community to climate change impacts. Most importantly, this control/command approach of resource grabbing has disconnected indigenous communities from their land, practice, and knowledge related to CC, and we have forgotten those natural prelaws (balance) due to the impact of colonisation’ (Datta et al. 2024) ‘We must consider the impacts to Indigenous culture and Indigenous women when climatic and environmental changes create barriers to cultural reproduction, a labor disproportionately borne by women.’ (Liddell et al. 2024)

## Appendix D Pathways Toward Gender Justice

**Table:**

Subtheme	Subcategory(ies)	Description	Key quotes and examples
Governance and decision making (*n* = 18)	Distributive governance	Shifting governance models was identified as potential means to promote pathways toward more equitable change and outcomes.	‘The idea of multi‐level governance is also necessary that will bring critical stakeholders such as civil society and Community Development Association into the fray to offer help that could alleviate the vulnerability of coastal women to erosion and flooding.’ (Ojakorotu and Olajide 2018) ‘Including women in environmental decision‐making is of upmost importance’ (Austin et al. 2021)
	Women's leadership	The literature identified that investments in women's leadership, greater representation of women and integrating women in consultative processes is important.	‘We suggest that understanding and amplifying Indigenous women‐led climate change solutions can inform the development of targeted interventions that build on existing sources of resilience within Indigenous communities and beyond’ (Chapola et al. 2024)
			‘The topic of China's persistent gender inequality and patriarchal repression was, to our knowledge, never on the agenda in Tzu Chi's many reading seminars or in meetings organised by the women volunteers. However, the organisation did serve as a space where urban middle‐class women felt that they could play a more assertive and self‐fulfilling role as publicly engaged citizens.’ (Chen and Hansen 2022) ‘Therefore, engendering policy at higher levels in Iran and the formulation of the national CC policy involves broad‐based consultations with diverse stakeholders from gender‐sensitive livelihood locations, with feedback loops ensuring their voices are heard at every stage of the process.’ (Goli et al. 2023) ‘There is a need to recognise how climate change impacts gender inequality within KwaZulu‐Natal and to involve the poor, particularly women, in all stages of development and implementation of local adaptation plans and strategies if the strategies are to be empowering and responsive to their interests’ (Hlahla et al. 2022)
	Sharing power and decision making	Not only is representation of women needed in formal leadership and governance structures, improved power dynamics in households and domestic settings is also needed to support gender equity.	‘Within households, elder men are often tasked with these decisions and actions. In the absence of their husbands, the women in these women‐only organisations exercise their power collectively, and in ways to benefit the goals of the group. In several cases, the profits are offered as loans to more vulnerable members.’ (Wood et al. 2021) ‘After reaching a peak of almost 40% at the Conference of the Parties (COP) 24, women's representation in country delegations has been in decline. For instance, at COP27, women delegates accounted for 36% of all national Party delegates, which is better than the 35% at the previous year but lower than pre‐pandemic COPs.’ (Pinho‐Gomes and Woodward 2024) ‘Our work in Mali suggests that there are pathways to climate resilience, and the labor of women of all ages is essential to mitigating the harms posed by climate‐linked food insecurity. However, pathways to resilience are situated within power structures that vary according to gender, age and location. Young women are presently navigating a climate landscape that did not exist in previous generations.’ (Wood et al. 2021) ‘Is essential to encourage women to participate in relevant decision‐making committees on environmental management. The extension and knowledge enhancement on the environmental conservation culture should also be supported by strengthening participatory approaches’ (Ghasemi et al. 2021)
	Equality in policy	Not only is representation and women's leadership needed, but also reforms in policy that prioritise and promote gender equity.	‘Disasters are frequently considered to be urgent events which impact everyone equally. Our findings suggest that this is simply not the case. An approach to disaster recovery which fails to consider gendered human rights impacts will also likely fail to create opportunities for women's equal participation politically and economically.’ (Detraz and Peksen 2017) ‘Gender‐Inclusive Climate Policies: Develop policies that integrate gender perspectives in climate adaptation and disaster risk reduction, ensuring women's participation and addressing their specific needs for greater resilience and equity.’ (Detraz and Peksen 2017) ‘There is need for government, civil society and members of the communities to come up with positive action that can help coastal women to respond to climate change from their position of vulnerability.’ (Ojakorotu and Olajide 2018) ‘Differences among women are crucial in the intersectional approach, and as the studies have shown, organising women‐only groups is insufficient to ensure women's participation’ (Jannat and Kusakabe 2024)
Inclusion of women in climate adaptation and resilience building (*n* = 16)	Including women in disaster planning	Women must be included in measures that seek to build their integration and inclusion in disaster planning, adaptation and resilience buildings.	‘Developing a disaster management plan that is gender inclusive with decentralised mechanisms should be promoted, and more women should become involved in the formulation of policies and plans.’ (Puwajarernpong et al. 2023)
	First Nations and Indigenous women	First Nations wisdom, knowledge and traditions was identified as an important contribution to building climate resilience.	‘Their unique challenges underscore the need for targeted strategies that address the intersectionality of gender, culture, and environmental justice in confronting climate change impacts’ (Chapola et al. 2024) ‘The narratives shared by our co‐researchers highlight the pivotal role of Indigenous women's leadership and the recognition of Indigenous land rights in offering a distinct intersectional perspective to the discourse. This lens considers not only the ecological impacts but also the nuances of climate adaptation within Indigenous communities. By centering the voices of Indigenous women, we gain valuable insights into holistic approaches to addressing the climate crisis that prioritise community resilience and sustainable practices.’ (Datta et al. 2024) ‘In our research, we have developed a long‐standing relationship with many Indigenous communities, from whom we have learned valuable insights. Therefore, in our research, we were not considered as participants but co‐researchers as knowledge belonged to the community, and we, as researchers, are learners’ (Datta et al. 2024) ‘The decolonization of climate crisis meanings from Indigenous women's perspectives aligns with broader efforts toward reconciliation and respectful collaboration’ (Datta et al. 2024)
	Women's organisations	Women's organisations were identified as being helpful in providing the structural foundations for women‐led climate change adaptation and resilience.	‘The topic of China's persistent gender inequality and patriarchal repression was, to our knowledge, never on the agenda in Tzu Chi's many reading seminars or in meetings organised by the women volunteers. However, the organisation did serve as a space where urban middle‐class women felt that they could play a more assertive and self‐fulfilling role as publicly engaged citizens. Volunteers often talked about Tzu Chi as a life‐changing experience, something that helped them find meaning and purpose in life. The government needed to improve its environmental education in society, and this opened up a space for agency.’ (Chen and Hansen 2022) ‘Women's organisations have proven to be effective vehicles for improving food security and community‐ based management of natural resources. For example, self‐help groups can address and improve women's access to land use and tenure, water rights, livestock production, credit and financial services, markets and transportation, agricultural extension services, participation in decision‐ making and community development, improved capacity building that takes gender differences into account.’ (Goli et al. 2023) ‘Within this, female leaders of the Prik Tai Community Flood Preparedness Committee are involved in health care, surveying, and welfare. These are all low‐risk tasks related to the care of community members. The male leaders are all in warning and public relations roles, or involved with security, evacuation, and movement. Clearly, women are assigned responsibilities based on the original concept that women should work in reproductive areas.’ (Puwajarernpong et al. 2023)
Building inclusive communities (*n* = 13)	Access to government services	Improving women's access to government services, such as social protection schemes.	‘Any exposure to government extension services is likely to make a greater impact on the household head's perception of climate change if there are relatively more adult females or more literate females’ (Floro et al. 2019) ‘Social protection schemes can facilitate women's access to resources and increase their role as decision makers in the family, though these outcomes do not happen quickly.’ (Ghasemi et al. 2021)
	Building social networks	Further investments in establishing women's community groups: social networks, including communities of practice and community‐based women's organisations is needed to foster inclusive communities.	‘Other measures include facilitating the creation of social networks among women in the community so that they can help each other in times of need.’ (Obossou et al. 2023) ‘A community of practice to allow for sharing of ideas, knowledge, information and good practices.’ (Hlahla et al. 2022) ‘This is an illustrative example of how intertwined networks, overlapping economic and political positions, and family connections are essential to such initiatives. This kind of political‐socioeconomic networking is very familiar in China, but one easily overlooked dimension is the role of gender. In the case of Riverbend Village, the environmental initiatives were facilitated by a female village head who were able to draw upon resources made available partly due to her gender.’ (Chen and Hansen 2022) ‘It is required to establish or strengthen women's community‐based organisations in rural communities in order to strengthen women's social participation and provide the necessary conditions in families for supporting women's participation in social programs.’ (Ghasemi et al. 2021)
	Action on violence	Policy making to reduce violence against women must be addressed.	‘The results of this analysis further support the need to not only articulate the impact of climate change on women but also elevate the experiences and voices of women who are impacted. Policymakers must include women in decision‐making and collaborate with policymakers in the global south’ (Munala et al. 2023)
	Family dynamics	Shifting power systems within family structures to give women more agency in decision making, and improved social status; aswell as reducing the burden they carry for the effects of the TPC on caregiving, may all present better opportunities for women's climate resilience.	‘Research results also indicated that women's position in their family has a significantly positive effect on their empowerment and environmental conservation’ (Ghasemi et al. 2021) ‘In the context of bushfires, this means that mothers cannot control the amount of smoke their (un)born children inhale as this is not within human power alone.’ (Allen et al. 2022) ‘In both developing and developed worlds, men have still a great deal of power in many aspects of women's lives. In the public domain, men design and implement policies which may or may not be in the interest of women's priorities and their needs as religious leaders, judges, village head and so on’ (Ghasemi et al. 2021)
	Public health	Improving public health measures, including vulnerability assessments, improving infrastructure, increasing staffing and optimising training were identified as important factors toward gender‐responsive climate resilience.	‘Our results reveal that public health infrastructure of water, doctors, and schooling are important in reducing suffering from droughts’ (Austin et al. 2021) ‘Therefore, local governments need to invest more in poverty alleviation, involve both men and women in decision‐making, and mainstream gender into climate change responses so as not to exacerbate gender inequalities or vulnerabilities.’ (Hlahla et al. 2022) ‘Our study underlines the relevance of including a sex and gender dimension in the analysis of the impacts of heat on the population and advocates the development of gender‐based adaptation policies to extreme heat.’ (Puwajarernpong et al. 2023)
Improving access to knowledge and individual capacity building (*n* = 10)	Education	Some articles recognised the role of education and training in empowering women to have capacity for climate change resilience.	“Support programs for women that promote diversified and climate‐resilient practices, providing training and access to markets, to enhance economic stability and food security” (Das 2024) ‘Our results suggest that there is a need to empower farmers to gradually move to more capital‐intensive practices. Gender, in particular, significantly influenced the use of the improved crop production system in this set of variables. This suggests that CSA programs and policy interventions should focus on improving equal access to CSA information and empowering women in household decision‐making. This can be achieved by establishing policy measures that would enhance women's access to formal education and extension services.’ (Obossou et al. 2023) ‘Women leaders were given the opportunity to participate in training activities to develop their knowledge and skills in dealing with and managing disasters, as well as to participate in meetings to present problems and plans to deal with disasters. The role of women leaders is still related to their ability to care for community members, and they tend to take on low‐risk jobs’ (Puwajarernpong et al. 2023) ‘Women's knowledge and skills (including life skills, etc.) and their abilities and skills to apply this knowledge in the required situations can be achieved through formal or informal education and in‐service training.’ (Ghasemi et al. 2021) ‘This requires educational programs, legal, administrative and credit support, which should be evolved as a dynamic movement over time.’ (Ghasemi et al. 2021) ‘Our results suggest that there is a need to empower farmers to gradually move to more capital‐intensive practices. Gender, in particular, significantly influenced the use of the improved crop production system in this set of variables. This suggests that CSA programs and policy interventions should focus on improving equal access to CSA information and empowering women in household decision‐making.’ (Obossou et al. 2023)
Increasing financial freedom and autonomy (*n* = 9)	Work and payment	Recognising, reducing and compensating women for their work is critical in liberating them to have greater resilience.	‘Despite contributing significantly to the societal and community fabric, their work is not adequately recognised or compensated, nor is it factored into workforce statistics or policymaking considerations. This research underscores the need to confront these issues and provide appropriate recognition and support to women in these roles.’ (Puwajarernpong et al. 2023) ‘It exceeds the limit because women have to absorb a high workload, at both the household and community levels’ (Puwajarernpong et al. 2023) ‘This is in particular important when we consider the central role that women can play in implementing successful adaptation strategies. Our results highlight the importance of targeted adaptation policies that can build on the positive impacts of climate change in reducing gender pay gap in rural communities by enabling and empowering women to use this opportunity for investing in their education, and independent smallholder farming practices.’ (Shayegh and Dasgupta 2024)
	Financial stability	Recognising and responding to women's poverty and/or financial instability is critical in helping to create opportunities where they can adapt to the impacts of climate change.	‘However, for women, ease of access to communication tools and facilities was the most important physical capital element. Given that most men own these assets, and women have less access to them, it is recommended that (1) the government commit to improving access to these assets and use environmentally friendly technologies as the most critical method of managing the impacts of CC to provide significant benefits to all, (2) villagers' ability to own new equipment and machinery be improved as a highly effective method of enhancing the physical capital of male and female rice farmers, and (3) sub‐categories such as access to credit (loans) and insurance for agricultural products through public and private bodies' support, free of any gender discrimination, be emphasised.’ (Goli et al. 2023) ‘Identify collectives as part of a response to climate change, the associations were viewed as promoting resilience, and sources of support for women who need additional resources to meet the needs of their households.’ (Wood et al. 2021) ‘There must be guidelines set for providing policy assistance related to the promotion of various welfare schemes for the elderly and their families.’ (Puwajarernpong et al. 2023) ‘As stated by the interviewed men, the most important financial assets are access to credit (loans) and insurance for agricultural products and producing revenue from agricultural activities whereas, for the women, access to credit (loans) and insurance for agricultural products are the most important assets. Therefore, (1) increasing the commitment level of guaranteed agricultural insurance for all farmers, (2) enhancing women's economic empowerment and improving their market access during the harvest season, and (3) empowering women by providing them with environmentally friendly technology and deploying home industries to create employment opportunities will be helpful in preventing or minimising damage to the livelihoods of female‐and male‐headed households.’ (Goli et al. 2023)
	Property rights	Improving the rights of women to purchase and own their own home and land not only has the potential to foster better land stewardship practices, but to improve their access to capital that builds their stability.	‘We find that women's immovable property rights are related to forest loss. This finding suggests that when governments protect the rights of women, the benefits of doing so extend far beyond women themselves. Given the health benefits of forests and the extent to which humans depend on forests’ (Sommer et al. 2022) ‘Toward this end, we can use our findings to guide policy that not only ensures the rights of women but also decreases forest loss and improves other aspects of women's lives at the same time’ (Sommer et al. 2022) ‘These community plots are managed by collectives of elder women, who collaboratively make decisions regarding land management, cultivation, selling crops and reinvesting money into their communities. Elder women also earn money off of the farm, often through selling shea butter. Earnings are then used to purchase additional food or seasonings’ (Wood et al. 2021) ‘All the interviewees said that owning the land made a difference. One reason was the ability to decide what to do with the land, … Another reason was the positive effect of the care and management of the land, which households only invested in once the land was theirs’ (Segnestam 2017) ‘Another advantage mentioned was that ownership of land made access to credits possible, since land was required as collateral’ (Segnestam 2017)
Healthcare workers	Factors for healthcare workers to consider	Healthcare workers can use contemporary research to improve the gender‐sensitivity of their work.	‘Local healthcare providers, both formal and informal, interviewed across the region said that this [food insecurity] was having a major impact on the nutrition of the children (Table 2). “Along with fish, there is now no milk and eggs for them. Neither are pulses and lentils growing locally. Thus, protein deficiency is acute among children and anaemia in young women,”’ (Ghosh and Dutta 2024) ‘Case study 2 is a regional women's health service with a strategic priority to identify the impact of climate change on women. The initiatives fall within the health promotion portfolio, and focus on advocacy and research in the area of climate change.’ (Patrick and Capetola 2011) ‘For teenagers, there must be a responsible adult at home to closely monitor the child. For adult women, we need to ensure that they do not become dependent on these or don't indulge in deliberate self‐harm, the rate of which is already very high,” said a doctor (MBBS, not a licensed psychiatrist) in Namkhana rural hospital. There is no psychiatrist in rural hospitals and MBBS doctors are not well‐trained to prescribe such medications. “It is also critical that their general health, particularly reproductive health, is not jeopardised,” said the lone psychiatrist in the largest government hospital of the region in Canning. However, he admitted that these medicines have penetrated rural areas with the collusion of a section of distributors, pharmaceutical companies and local quacks, who feel obligated to help women in distress’ (Ghosh and Dutta 2024)

## Appendix E Outcomes

**Table:**

Subtheme	Subcategory(ies)	Description	Key quotes and examples
Women's leadership influence (*n* = 8)	Adoption of environmental policies	Investing in women's participation in governance leads to the adoption of more climate‐considerate policies.	‘The results show that the coefficient of WIP [women in parliament] is positive and statistically significant, indicating that a higher proportion of women in parliament indeed leads to a more favourable adoption of climate change policies. Conversely, the coefficient of corruption is negative, suggesting that higher levels of corruption hinder the adoption of such policies.’ (Dutta et al. 2024) ‘Overall, it is judged that female executives in top management teams are giving a positive influence to companies in the long‐term by improving the disclosure environment with a new perspective. In order to secure long‐term growth with improved disclosure policy, companies need to increase the proportion of female executives in the organisation.’ (Eunsoo Kim 2022) ‘In general, the protection of natural resources, preservation of forest cover, conservation of water, promotion of sustainable agriculture with reduced chemical usage, implementation of scientifically sound sanitation practices, and broader measures to counteract global warming emerge as important agendas consistently championed and debated by women representatives.’ (Dutta et al. 2024) ‘Women (*X* ^−^ = 16.5) used CCAS [climate change adaptation strategies] more than men (*X* ^−^ = 14/8). Therefore, women play a more prominent role in CCA behaviours.’ (Goli et al. 2023) ‘Female political representation in national parliaments can also influence climate change policy outcomes by leading to the adoption of strong environmental protection policies’ (Asongu et al. 2022)
	Women's perspectives	Including women's perspectives in decision‐making can lead to reduced societal vulnerability to climate change impacts.	‘Women's political participation enhances economic, social, and government readiness, which in turn reduce vulnerability to climate change’ (Asongu et al. 2022) ‘The current study shows that affection toward traditional diets is significant and associated with gender norms in a traditional community. Further, due to some genderdefined norms of the traditional communities, gender‐inequality is evident. Hence, integrating women's perspectives could ensure nutritional security and aid in gender‐inclusive adaptation strategies in the Himalayan Region of India’ (Das 2024)
	Gender equity	Improvements toward gender equity foster more climate resilient systems.	‘Countries around the world that promote women's empowerment are indirectly improving their resilience to climate change’ (Asongu et al. 2022)
Finance and work (*n* = 7)	Financial resources	Investments in financial freedom for women can lead to both empowerment of women and increases in environmental conversation.	‘The efforts made in the last two decades to provide the necessary conditions for women's participation in rural councils, women's micro‐credit funds and rural women's production cooperatives have been effective for both empowerment and environmental conservation’ (Ghasemi et al. 2021) ‘Rural women with earned income are likely to have a better intra‐household bargaining position, thus increasing the areas open for consultation and negotiation between husbands and wives’ (Floro et al. 2019)
	Changes in labour force	Climate change may lead to increases in supply of low‐skilled male labour to be available for work. If the supply of female labour remains stable or does not increase as much, their labour becomes relatively more scarce.	‘However, as the supply factor ratio of low‐skilled male to female labour increases due to climate change (panel (b)), the relative wages of low‐skilled female labour increases’ (Shayegh and Dasgupta 2024)
	Property rights	Property rights for women may not only have positive impacts on women's ability to recover from climate change related impacts—but also leads to better environmental outcomes.	‘First, we find support for our main hypothesis that higher levels of women's property rights correspond with less forest loss.’ (Sommer et al. 2022) ‘We find that women's immovable property rights are related to forest loss. This finding suggests that when governments protect the rights of women, the benefits of doing so extend far beyond women themselves.’ (Sommer et al. 2022)
Education (*n* = 2)		Improvements to women's access to education may yield further opportunities to build climate literacy.	‘Adding to their traditional practices of waste collection and recycling (in addition to the charity work that remains at the core of Tzu Chi's work), the organisation in Suzhou has now opened a museum for science‐based communication about climate change, and it has organised an increasing amount of teaching for school classes about both climate change and more practical ways of dealing with waste collection, sorting, and recycling. In one of the branches we visited in 2020, volunteers had given 40 training classes to a total of around 2500 students from primary to secondary high school, just within the year of 2019.’ (Chen and Hansen 2022) ‘Model 5 GME estimates show that FH with access to government extension are 30% more likely to perceive climate change if there are relatively more educated female members’ (Floro et al. 2019) ‘The presence of literate females in the households can help in the processing of information obtained from government extension services, and thus increases the head's awareness of climate change.’ (Floro et al. 2019)
